# Enhanced regulation of prokaryotic gene expression by a eukaryotic transcriptional activator

**DOI:** 10.1038/s41467-021-24434-9

**Published:** 2021-07-05

**Authors:** I. Cody MacDonald, Travis R. Seamons, Jonathan C. Emmons, Shwan B. Javdan, Tara L. Deans

**Affiliations:** Department of Biomedical Engineering, University of Utah, Salt Lake City, UT USA

**Keywords:** Genetic engineering, Synthetic biology, Prokaryote

## Abstract

Expanding the genetic toolbox for prokaryotic synthetic biology is a promising strategy for enhancing the dynamic range of gene expression and enabling new engineered applications for research and biomedicine. Here, we reverse the current trend of moving genetic parts from prokaryotes to eukaryotes and demonstrate that the activating eukaryotic transcription factor QF and its corresponding DNA-binding sequence can be moved to *E. coli* to introduce transcriptional activation, in addition to tight off states. We further demonstrate that the QF transcription factor can be used in genetic devices that respond to low input levels with robust and sustained output signals. Collectively, we show that eukaryotic gene regulator elements are functional in prokaryotes and establish a versatile and broadly applicable approach for constructing genetic circuits with complex functions. These genetic tools hold the potential to improve biotechnology applications for medical science and research.

## Abstract (web summary)

Expanded toolkits for prokaryotic synthetic biology can enhance the dynamic range of gene expression. Here the authors move the eukaryotic transcription factor QF into *E. coli* and integrate it into genetic devices.

## Introduction

The field of synthetic biology has revolutionized how cells can be reprogrammed through the assembly of genetic parts, or modules, into more complex genetic circuits. This approach is broadly applicable to many organisms and provides a means to control cell behavior in new and predictable ways^1–13^. To accomplish this, genetic parts are often taken from diverse organisms and used to establish orthogonal, independent functions within cells^14,15^. Orthogonal control elements of genetic circuits do not exist naturally in the host organism and as a result are unlikely to interfere with, or be compromised by, host signaling events. Genetic parts are traditionally moved from prokaryotic organisms to more complex eukaryotic cells to provide orthogonal gene control in higher organisms^16–21^; however, genetic parts from eukaryotes are rarely, if ever, moved from eukaryotes to prokaryotes.

Transcription factors are DNA-binding proteins that repress or activate transcription by binding to a specific DNA sequence within the genome, and they have been widely used to build genetic circuits that regulate gene expression. In prokaryotes, repressor proteins are predominately used to control gene expression because prokaryotic genes are on by default^22^. Most prokaryotes have a single RNA polymerase (RNAP) that recognizes all of the promoters and transcribes all the RNA in the organism unless repressor proteins block RNAP from binding to the promoter sequence. Inducible transcription in bacteria is most commonly achieved by de-repression, or the removal of repressor proteins, to enable the binding of RNAP and the transcription of downstream gene(s)^23^.

The bacteriophage T7 promoter and corresponding T7 RNA polymerase (T7RNAP) are widely used in bacteria for high recombinant protein production^24^. Since the endogenous RNAP does not bind to the T7 promoter, the system provides orthogonal control of gene expression in prokaryotes. Because T7RNAP is highly selective for the T7 promoter, transcription of downstream genes is also selective^25^. This system can be made inducible by controlling the transcription of *T7RNAP* using bacterial systems such as AraC^26^, LacI^27^, and TetR^14^ and placing their respective DNA-binding sites upstream of an endogenous promoter that drives the expression of *T7RNAP*. The repressor proteins of these systems bind to their respective DNA-binding sites to prevent the transcription of *T7RNAP*. In the presence of their small molecule inducer, arabinose, isopropyl β-D-1-thiogalactopyranoside (IPTG), and anhydrotetracycline (aTc), respectively, the repressor proteins become dislodged from their binding sites, enabling endogenous RNAP to bind and transcribe *T7RNAP*. To date, the inducible T7 promoter is the most widely used orthogonal system in prokaryotes for high levels of recombinant protein production^28^.

We propose an alternative approach to orthogonal regulation of gene expression in prokaryotes that investigates circuits regulated by transcriptional activators rather than baseline repression. To accomplish this, we reversed the conventional approach of moving gene regulatory parts from prokaryotes to eukaryotes by moving the activating eukaryotic transcription factor *QF* and its corresponding DNA-binding site, QUAS, from the filamentous fungus *Neurospora crassa* to bacteria. When glucose levels are low, the fungus utilizes the regulatory genes from its quinic acid (QA) gene cluster (*qa*) to use quinic acid as a carbon source^29,30^. The *qa* gene cluster includes a gene encoding the transcription factor *QF*. QUAS is found upstream of the *qa* gene cluster and other *QF* regulated genes. QF is a transcriptional activator that promotes the transcription of downstream genes when it binds to QUAS. The *qa* gene cluster also encodes the negative regulator QS that prevents QF from binding to the transcriptional machinery at its activation domain preventing the transcription of downstream genes. Transcriptional repression can be reversed with the addition of quinic acid (Supplementary Fig. 1). The interactions of QF, QUAS, and QS are collectively referred to as the Q system. A second generation of the *QF* transcription factor (*QF2*) that has the middle region of the protein removed to decrease toxicity in other organisms was used in this study^31^.

Various components of the Q system have been successfully applied to regulate gene expression in *Drosophila*^31–33^, cultured mammalian cells^34,35^, zebrafish^36^, and *Caenorhabditis elegans* (*C. elegans*)^37^. To test whether *qa* clusters of genes function in prokaryotes, we moved the gene encoding the *QF* transcription factor and its QUAS binding site to expression plasmids specific for bacteria. We found that the QUAS binding domain introduced robust transcriptional activation in the presence of QF when placed downstream of the T7 promoter. In contrast, when the QUAS binding domain was placed upstream of the T7 promoter, tight transcriptional repression in the absence of QF was observed. Moreover, we demonstrate the versatility of this genetic tool in bacteria by showing its compatibility with the TetR system, and show that it has a broad dynamic range of regulated gene expression. Using the eukaryotic Q system, we present alternative strategies for controlling gene expression in bacteria that expand the genetic toolbox in prokaryotic synthetic biology and enhances the production capabilities of mRNA and proteins for biotechnology applications.

## Results

### The position of QUAS relative to the T7 promoter enables switch-like gene expression

**Fig. 1:**
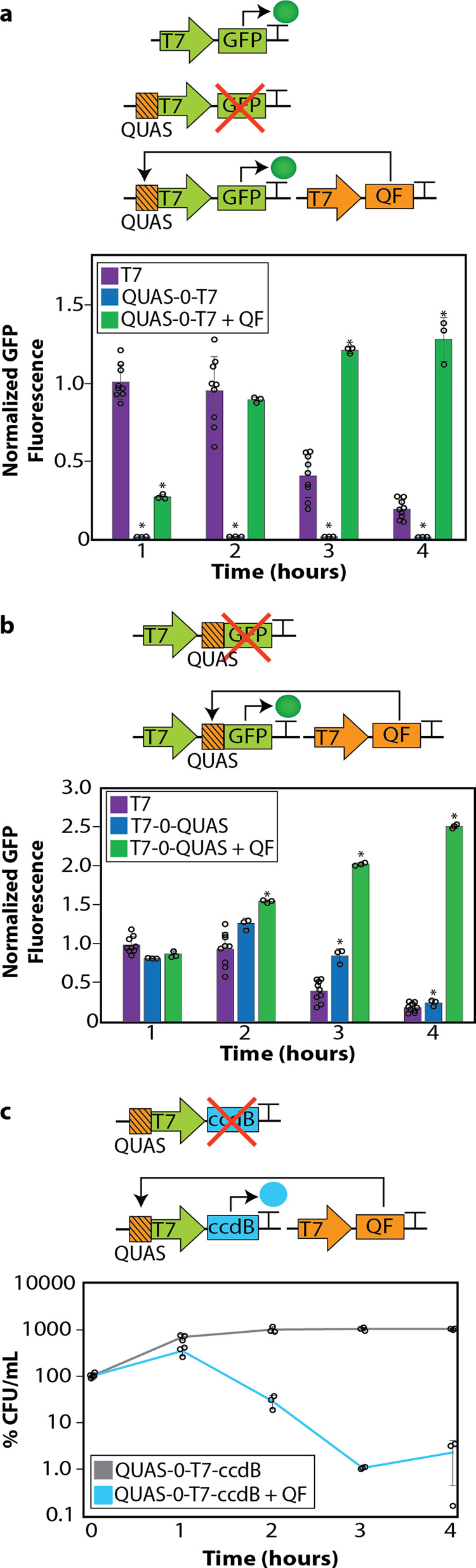
The Q system in bacteria. **a** Schematic of the control plasmid with the bacteriophage T7 promoter constitutively expressing *GFP* followed by a T7 terminator sequence (T) (top). Schematic of placing QUAS (orange square with lines) directly upstream of the T7 promoter (QUAS-0-T7) driving the expression of *GFP* (green) (middle). Schematic of adding the constitutive expression of *QF* (orange) to the system (QUAS-0-T7 + QF) (bottom). When QF is present it binds to QUAS and activates the expression of *GFP* (green). Flow cytometry quantifying the GFP fluorescence over a 4-h period after the initial induction of 0.5 mM IPTG (added at time zero). A two-tailed *t*-test was performed to determine statistical significance (*P* < 0.01) between the T7 control and components of the Q system upstream of the T7 promoter. An aster (*) represents statistical significance. **b** Schematic of QUAS placed directly downstream of the T7 promoter (T7-0-QUAS) driving the expression of *GFP* (top). Schematic of adding the constitutive expression of *QF* to the system (bottom). When QF is present it binds to QUAS and activates the expression of *GFP* (T7-0-QUAS + QF). Flow cytometry quantifying the GFP fluorescence over a 4-h period after the initial induction of 0.5 mM IPTG (added at time zero) to initiate the transcription of *T7RNAP* to be available for transcribing genes downstream of the T7 promoters. A two-tailed *t*-test was performed to determine statistical significance (*P* < 0.01) between the T7 control and components of the Q system downstream of the T7 promoter. An aster (*) represents statistical significance. **c** Schematic controlling *ccdB* expression by replacing *GFP* with the toxin *ccdB* (turquoise) with the QUAS directly upstream of the T7 promoter (top) and adding the constitutive expression of *QF* (orange) to the system (bottom). When QF is present it binds to QUAS and activates the expression of the *ccdB* toxin. Percent colony forming units per milliliter (%CFU/mL) over a 4-h period after induction of 0.5 mM IPTG (added at time zero) to initiate the transcription of *T7RNAP* in the absence of QF (gray line) and the presence of QF (blue line). In all experiments, IPTG was added to initiate transcription of *T7RNAP*, allowing transcription of T7-controlled genes. Each experiment consisted of generating data from at least three separate bacterial colonies grown in overnight cultures, where circles represent individual data points in the plots. These experiments were repeated independently at least three times with similar results. The geometric mean of each sample was calculated via FlowJo, and error bars indicate standard deviation. Fluorescence values were normalized to the T7 control expression at 1 h after IPTG induction. The error bars indicate 95% confidence intervals of the mean of fluorescence, and data are presented as mean ± standard deviation. Source data are available as a Source data file.

To investigate whether QF could mediate orthogonal gene control in prokaryotes, the 16 base pair QUAS DNA sequence was placed directly upstream of a T7 promoter (QUAS-0-T7) driving the expression of *GFP* (Supplementary Table 2). The construct was tested in the BL21(D3) strain of *E. coli*. This bacterial strain transcribes *T7RNAP* upon induction with IPTG. Unless otherwise stated, all experiments presented are in the presence of 0.5 mM IPTG to induce the transcription of *T7RNAP*. We placed a degradation tag on the C-terminal end of GFP to limit its half-life and enable more accurate reporting of gene expression dynamics in our circuits^38–40^. In the absence of QUAS, the T7 promoter actively transcribes *GFP* following *T7RNAP* transcription with IPTG induction (Fig. 1a, top construct). However, inserting a single QUAS binding site directly upstream of the T7 promoter prevents *GFP* expression despite the presence of IPTG and induction of *T7RNAP* (Fig. 1a, middle construct; b top construct), with GFP fluorescence equivalent to the background fluorescence level of untransformed cells (Supplementary Fig. 2). When QF is introduced in a separate plasmid, the QUAS-0-T7 system is activated and expression of *GFP* can be detected within the first hour. Three hours after IPTG induction, GFP fluorescence surpassed the level from the T7 promoter control (Fig. 1a). Thus, when T7RNAP is present upon IPTG induction, the eukaryotic transcription factor QF can activate gene expression in bacteria beyond baseline levels. The QUAS-0-T7 circuit has switch-like behavior with a tight off state and robust activation of *GFP* expression that was dependent on the expression of *QF*.

Since *cis*-regulatory elements in eukaryotes can function upstream and downstream of promoters, we evaluated the impact on gene expression of placing QUAS downstream of the T7 promoter (T7-0-QUAS) (Fig. 1b). Repositioning QUAS resulted in *GFP* expression similar to that of the T7 control (Fig. 1b) with both circuits reaching their peak *GFP* expression in the absence of QF 2 h after IPTG induction of *T7RNAP*. For both circuits, expression quickly diminished 3 h after of *T7RNAP* induction and reached baseline expression by 4 h. In contrast, *GFP* expression from the T7-0-QUAS system in the presence of QF was greater starting at hour two when compared to the T7-0-QUAS without QF and the T7 control (Fig. 1b). Together these experiments demonstrated that QUAS can function on either side of the transcriptional start site in bacteria. Moreover, placing QUAS downstream of the T7 promoter enabled higher and more sustained *GFP* expression in the presence of QF than when QUAS was placed upstream of the T7 promoter; though, baseline expression was not blocked and resembled the T7 control (Fig. 1a, b).

### Upstream placement of QUAS tightly represses gene expression

Placement of QUAS upstream of the T7 promoter prevented *GFP* expression despite the presence of T7RNAP after the addition of IPTG (Fig. 1a, b ). To further evaluate the extent of T7 promoter silencing, *GFP* was replaced with *ccdB* (Fig. 1c), a highly toxic protein in the CcdB/CcdA toxin/antitoxin system that causes severe DNA damage and cell death^41–45^. We demonstrated that *E. coli* cells containing QUAS-0-T7-*ccdB* constructs continue to grow over a 4-h time period without growth inhibition or cell death, presumably due to the tight off state that QUAS provides (Fig. 1c and Supplementary Fig. 3). In contrast, QF activated expression of *ccdB* from QUAS-0-T7, causing 99% cell death within 2 h of induction of *T7RNAP* (Fig. 1c). We conclude that placing QUAS upstream of the T7 promoter establishes a tightly regulated switch that can maintain tight off states and rapidly activates in the presence of QF.

### QUAS spacing upstream of the T7 promoter impacts gene expression

**Fig. 2:**
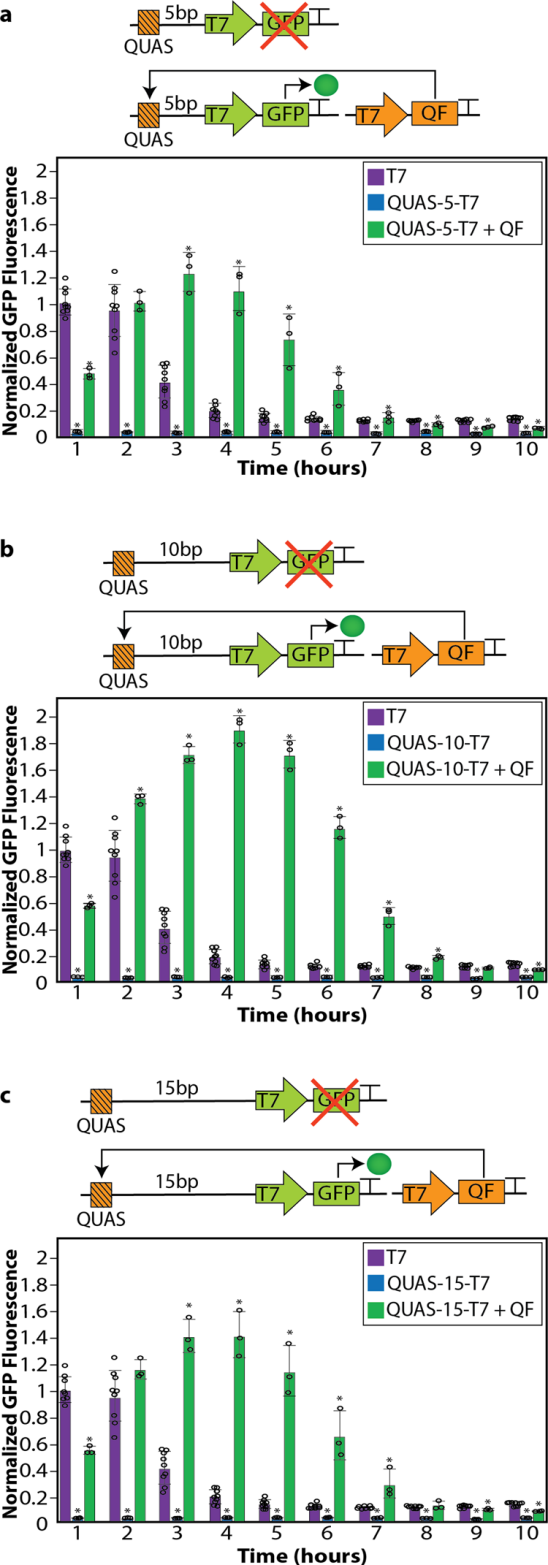
QUAS spacing upstream of the T7 promoter. **a** Schematic depicting QUAS (orange square with lines) spaced 5 base pairs upstream of the T7 promoter (QUAS-5-T7) driving the expression of *GFP* (green) (top) and the effect of QF (orange) on *GFP* expression (bottom). In the absence of QF, the transcription of *GFP* is not initiated. When QF is present, (QUAS-5-T7 + QF) it binds to QUAS and activates the transcription of *GFP*. Flow cytometry quantifying GFP fluorescence over a 10-h period after the initial induction of 0.5 mM IPTG (added at time zero) to initiate the transcription of *T7RNAP* without QUAS (purple), with QUAS 5 base pairs upstream of the T7 promoter in the absence (blue), and presence (green) of QF. A two-tailed *t*-test was performed to determine statistical significance (*P* < 0.02) between the T7 control and components of the Q system with QUAS placed 5 base pairs upstream of the T7 promoter. An aster (*) represents statistical significance. **b** Schematic depicting QUAS (orange square with lines) spaced 10 base pairs upstream of the T7 promoter (QUAS-10-T7) driving the expression of *GFP* (green) (top) and the effect of QF (orange) on *GFP* expression (bottom). In the absence of QF the transcription of *GFP* is not initiated, however, in the presence of QF (QUAS-10-TF + QF) it binds to QUAS and activates the transcription of *GFP*. Flow cytometry quantifying *GFP* fluorescence over a 10-h period after the initial induction of 0.5 mM IPTG (added at time zero) to initiate the transcription of *T7RNAP* without QUAS (purple), with QUAS 10 base pairs upstream of the T7 promoter in the absence (blue) and presence (green) of QF. A two-tailed *t*-test was performed to determine statistical significance (*P* < 0.002) between the T7 control and components of the Q system with QUAS placed 10 base pairs upstream of the T7 promoter. An aster (*) represents statistical significance. **c** Schematic depicting QUAS (orange square with lines) spaced 15 base pairs upstream of the T7 promoter (QUAS-15-T7) driving the expression of *GFP* (green) (top) and the effect of QF (orange) on *GFP* expression (bottom). In the absence of QF the transcription of *GFP* is not initiated. When QF is present, (QUAS-15-T7 + QF) it binds to QUAS and activates the transcription of *GFP*. Flow cytometry quantifying GFP fluorescence over a 10-h period after the initial induction of 0.5 mM IPTG (added at time zero) to initiate the transcription of *T7RNAP* without QUAS (purple), with QUAS 15 base pairs upstream of the T7 promoter in the absence (blue), and presence (green) of QF. A two-tailed *t*-test was performed to determine statistical significance (*P* < 0.007) between the T7 control and components of the Q system with QUAS placed 15 base pairs upstream of the T7 promoter. An aster (*) represents statistical significance. Each experiment consisted of generating data from at least three separate bacterial colonies grown in overnight cultures, where circles represent individual data points in the plots. These experiments were repeated independently at least three times with similar results. The geometric mean of each sample was calculated via FlowJo, and error bars indicate standard deviation. Fluorescence values were normalized to the T7 control (purple) expression at 1 h. The error bars indicate 95% confidence intervals of the mean of fluorescence, and data are presented as mean ± standard deviation. Source data are available as a Source data file.

For other upstream activation sequences, the position of the sequence relative to the promoter influences the efficacy of transcription initiation^46^. To determine whether the location of QUAS upstream of the T7 promoter influences *GFP* gene expression, QUAS was placed at various positions upstream of the T7 promoter (Fig. 2). Because one half-turn of the DNA double-helix is ~5 base pairs, the placement of QUAS was shifted in intervals of 5 base pairs, enabling the bound QF orientation to be rotated one half-turn. QUAS was placed 5, 10, or 15 base pairs upstream of the T7 promoter (Fig. 2 and Supplemental Table 2). These differ from QUAS-0-T7 where the QUAS and T7 sequences were adjacent. We investigated the modifications’ impact on the duration of gene expression by observing GFP fluorescence over a 10-h time period (Fig. 2). All three QUAS-spacing plasmids produced switch-like expression with tight off states in the absence of QF, and activation of gene expression in the presence of QF. Of the positions tested, the QUAS placed 10 base pairs upstream of the T7 promoter (QUAS-10-T7) produced the highest expression of *GFP* in the presence of QF (Fig. 2b). In addition, QUAS-10-T7 with QF maintained a robust level of expression for a longer period of time when compared to all other upstream locations (Fig. 2a, c).

### QUAS downstream of the T7 promoter enables amplification of gene expression

**Fig. 3:**
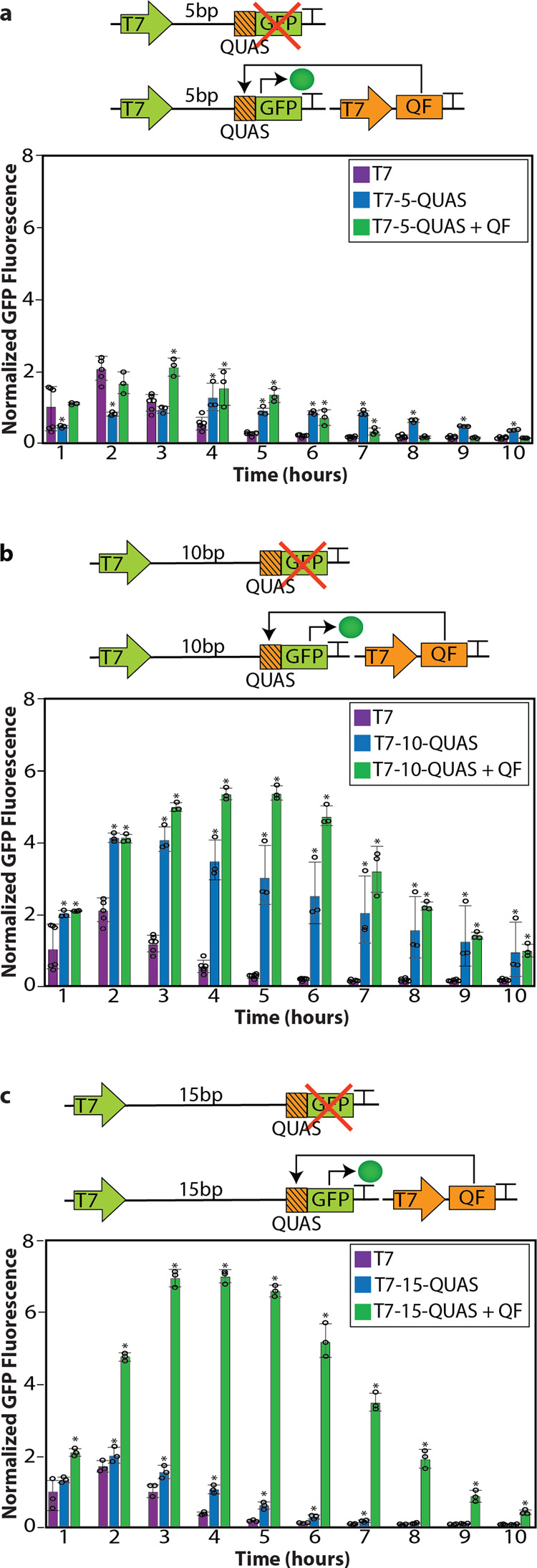
QUAS spacing downstream of the T7 promoter. **a** Schematic depicting the QUAS (orange square with lines) spaced 5 base pairs downstream of the T7 promoter (T7-5-QUAS) driving the expression of *GFP* (green) (top) and the effect of QF (orange) on *GFP* expression (bottom). In the absence of QF the transcription of *GFP* is not initiated. When QF if present (T7-5-QUAS + QF) it binds to QUAS and activates the transcription of *GFP*. Flow cytometry quantifying GFP fluorescence over a 10-h period after the initial induction of 0.5 mM IPTG (added at time zero) to initiate the transcription of T7RNAP without QUAS (purple), with QUAS 5 base pairs downstream of the T7 promoter in the absence (blue), and presence (green) of QF. A two-tailed *t*-test was performed to determine statistical significance (*P* < 0.04) between the T7 control and components of the Q system with QUAS placed 5 base pairs downstream of the T7 promoter. An aster (*) represents statistical significance. **b** Schematic depicting the QUAS (orange square with lines) spaced 10 base pairs downstream of the T7 promoter (T7-10-QUAS) driving the expression of *GFP* (green) (top) and the effect of QF (orange) on *GFP* expression (bottom). In the absence of QF the transcription of *GFP* is not initiated. When QF if present (T7-10-QUAS + QF) it binds to QUAS and activates the transcription of *GFP*. Flow cytometry quantifying GFP fluorescence over a 10-h period after the initial induction of 0.5 mM IPTG (added at time zero) to initiate the transcription of T7RNAP without QUAS (purple), with QUAS 5 base pairs downstream of the T7 promoter in the absence (blue), and presence (green) of QF. A two-tailed *t*-test was performed to determine statistical significance (*P* < 0.04) between the T7 control and components of the Q system with QUAS placed 5 base pairs downstream of the T7 promoter. An aster (*) represents statistical significance. **b** Schematic depicting QUAS (orange square with lines) spaced 10 base pairs upstream of the T7 promoter (QUAS-10-T7) driving the expression of *GFP* (green) (top) and the effect of QF (orange) on *GFP* expression (bottom). In the absence of QF the transcription of *GFP* is not initiated, however, in the presence of QF (QUAS-10-TF + QF) it binds to QUAS and activates the transcription of *GFP*. Flow cytometry quantifying *GFP* fluorescence over a 10-h period after the initial induction of 0.5 mM IPTG (added at time zero) to initiate the transcription of *T7RNAP* without QUAS (purple), with QUAS 10 base pairs upstream of the T7 promoter in the absence (blue) and presence (green) of QF. A two-tailed *t*-test was performed to determine statistical significance (*P* < 0.05) between the T7 control and components of the Q system with QUAS placed 10 base pairs upstream of the T7 promoter. An aster (*) represents statistical significance. **c** Schematic depicting the QUAS (orange square with lines) spaced 15 base pairs downstream of the T7 promoter (T7-15-QUAS) driving the expression of *GFP* (green) (top) and the effect of QF (orange) on *GFP* expression (bottom). In the absence of QF the transcription of *GFP* is not initiated. When QF if present (T7-15-QUAS + QF) it binds to QUAS and activates the transcription of *GFP*. Flow cytometry quantifying GFP fluorescence over a 10-h period after the initial induction of 0.5 mM IPTG (added at time zero) to initiate the transcription of *T7RNAP* without QUAS (purple), with QUAS 15 base pairs downstream of the T7 promoter in the absence (blue), and presence (green) of QF. A two-tailed *t*-test was performed to determine statistical significance (*P* < 0.03) between the T7 control and components of the Q system with QUAS placed 5 base pairs downstream of the T7 promoter. An aster (*) represents statistical significance. Each experiment consisted of generating data from at least three separate bacterial colonies grown in overnight cultures, where circles represent individual data points in the plots. These experiments were repeated independently at least three times with similar results. The geometric mean of each sample was calculated via FlowJo, and error bars indicate standard deviation. Fluorescence values were normalized to the T7 control (purple) expression at 1 h. The error bars indicate 95% confidence intervals of the mean of fluorescence, and data are presented as mean ± standard deviation. Source data are available as a Source data file.

Since the relative position of QUAS upstream of T7 influenced expression, we also investigated the impact of positioning QUAS at different downstream locations relative to the T7 promoter. Similar to the upstream placements, QUAS was placed at 5, 10, and 15 base pairs downstream of the T7 promoter (Fig. 3). In all cases, we kept the distance between the end of the QUAS and the ribosome binding site (RBS) the same in addition to the distance between the RBS and start codon of the downstream gene (Supplementary Table 2). The relative position of the QUAS insertion impacted the intensity and duration of *GFP* expression. This position-dependent effect was accentuated with the addition of QF, which resulted in the highest and most sustained levels of expression from T7-15-QUAS. Expression from the T7-15-QUAS + QF construct lasted 6 h longer than the T7-15-QUAS and T7 controls (Fig. 3). While our flow cytometry results indicate that the bacteria maintain fairly homogeneous populations throughout this time course, after 6 h of IPTG induction of *T7RNAP* we could detect heterogeneity in the T7-0-QUAS and T7-0-QUAS + QF populations of bacteria with the emergence of a sub-population with reduced GFP fluorescence (Supplementary Fig. 4). This is likely due to the cells entering the stationary phase, where they stop producing GFP (Supplementary Fig. 6). Due to the degradation tag on GFP, we observe rapid degradation of fluorescence once it enters this growth phase (Supplementary Fig. 4). Despite this, the majority of the bacteria-containing T7-0-QUAS and T7-0-QUAS + QF maintained *GFP* expression over the course of the 10-h experiment compared to the controls. Overall, the bacteria with T7-0-QUAS and T7-0-QUAS + QF had higher levels of *GFP* expression per cell than T7-*GFP* control cells (Supplementary Fig. 4).

### Design of activator-repressor based genetic circuits

**Fig. 4:**
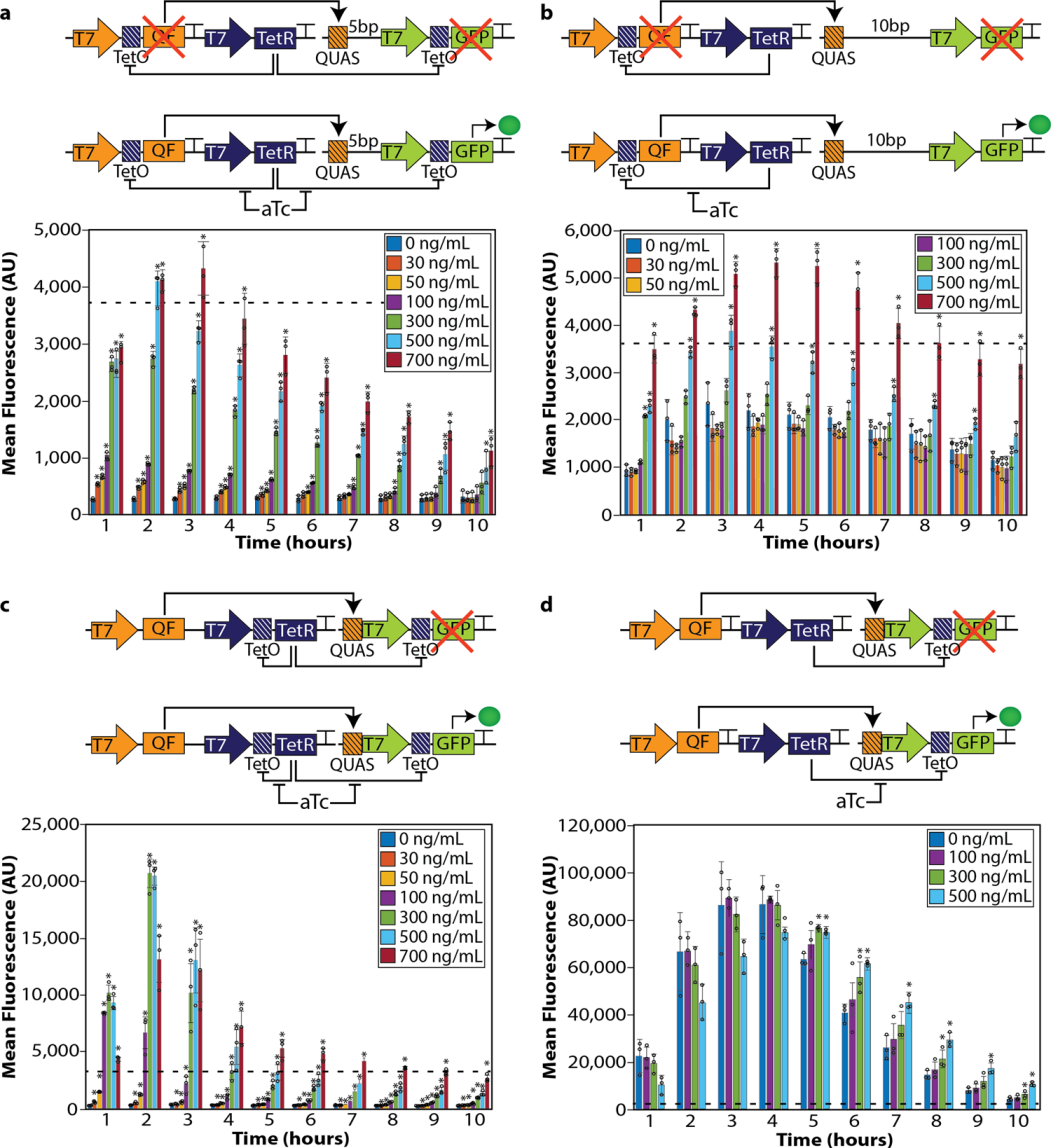
Genetic devices built with components from the Q system coupled with the TetR system. **a** Biological sensor with QUAS (orange box with lines) placed 5 base pairs upstream of the T7 promoter driving the expression of *GFP* (green). A TetO site (blue square with lines) was placed upstream of GFP and QF. The *tetR* gene is constitutively expressed by a T7 promoter (blue arrow). Schematic in the absence of aTc (top) indicates that both the expression of *QF* (orange rectangle) and *GFP* (green rectangle) is repressed by TetR proteins since T7RNAP cannot bind. Schematic when aTc is added to the system (bottom), the TetR proteins no longer prevent T7RNAP from binding, and transcription of *QF* and *GFP* turns on. Flow cytometry quantifying the amount of *GFP* expression with varying amounts of aTc. **b** Biological sensor with QUAS (orange box with lines) placed 10 base pairs upstream of the T7 promoter driving the expression of GFP (green). The *tetR* gene (blue) is constitutively expressed by a T7 promoter (blue). Schematic in the absence of aTc indicates that both the expression of *QF* and *GFP* is repressed by TetR proteins (top). Schematic when aTc is added to the system, the TetR proteins no longer prevent T7RNAP from binding, and transcription of *QF* and *GFP* turns on (bottom). Flow cytometry quantifying the amount of GFP expression with varying amounts of aTc. **c** Low pass sensor. In this design, the T7 terminator sequence between the *QF* and *tetR* genes was removed to produce more TetR proteins. QUAS was placed directly upstream of the T7 promoter driving *GFP* expression. A TetO site is located upstream of both the *GFP* and *tetR* genes so they can be regulated by aTc. Schematic of the low pass sensor in the absence of aTc (top). Schematic of the low pass filter in the presence of aTc (bottom). Flow cytometry quantifying the amount of *GFP* expression with varying amounts of aTc. **d** Genetic device to produce large quantities of protein. This genetic circuit has QUAS placed directly upstream of the T7 promoter driving the expression of *GFP*. A TetO site was placed upstream of *GFP* to regulate its expression with aTc. In this circuit, the *QF* is constitutively expressed and binds to QUAS. Schematic in the absence of aTc, TetR binds to the TetO sites to repress the expression of *GFP* (top). Schematic when aTc is added to the system, the TetR proteins no longer bind, enabling the T7RNAP to bind and transcribe *GFP* (bottom). Flow cytometry quantifying *GFP* expression with varying amounts of aTc. In all experiments, the dotted line indicates the maximum expression of the traditional TetR system without the Q system (~3700 arbitrary units). GFP was quantified for all constructs using flow cytometry over a 10-h period after the initial induction of 0.5 mM IPTG (added at time zero to initiate transcription of *T7RNAP*, allowing transcription of T7-controlled genes. Each experiment consisted of generating data from at least three separate bacterial colonies grown in overnight cultures, where circles represent individual data points in the plots. These experiments were repeated independently at least three times with similar results. A two-tailed *t*-test was performed to determine statistical significance (*P* < 0.05) between the 0 ng/mL of aTc with each induction amount over 10 h. An aster (*) represents statistical significance. The geometric mean of each sample was calculated via FlowJo, and error bars indicate standard deviation. The error bars indicate 95% confidence intervals of the mean of fluorescence, and data are presented as mean ± standard deviation. Source data are available as a Source data file.

To generate high-performance of programmable bacteria with enhanced regulated production of mRNA and protein, we coupled QF and its QUAS DNA-binding site with the TetR system to create a collection of genetic devices that mediated specific responses to aTc. Unlike other regulatory systems, the unique position-dependent characteristics of QUAS in bacteria makes this a highly versatile and modular component for custom-made genetic devices. The response characteristics of these circuits were evaluated relative to the traditional TetR system using a graded series of aTc concentrations over a 10-h culture period (Supplementary Fig. 5). For the analysis of our devices, each circuit was compared to the maximum expression of *GFP* attained through the activation by the traditional TetR system, which is represented by a dashed line (Fig. 4). We sought to design genetic devices that were off in the uninduced state, therefore each design of these devices contains a QUAS sequence positioned upstream of the T7 promoter to take advantage of the tight off state created by this configuration.

Placing QUAS 5 base pairs upstream of the T7 promoter driving the expression of *GFP* produces a biosensor similar to the traditional TetR system (Fig. 4a and Supplementary Fig. 5). In this design, the TetR repressor proteins bind to the *tetO* sites located in two places: downstream of the T7 promoter driving the expression of QF and downstream of the T7 promoter driving the expression of *GFP*, repressing the transcription of both *QF* and *GFP* (Fig. 4a, top circuit). The addition of aTc produces a conformational change in the TetR proteins, causing them to no longer bind to the TetO sites. T7RNAP is then permitted to bind to the promoter to transcribe *QF* and *GFP* (Fig. 4a, bottom circuit). This circuit design enables higher *GFP* expression compared to the traditional TetR system (Fig. 4a, dotted line and Supplementary Fig. 5). Similar to the traditional TetR system, lower concentrations of aTc (≤100 ng/mL) could be distinguished within the first 4 h; however, beyond 4 h these lower concentrations were indistinguishable, and only the higher concentrations of aTc (≥300 ng/mL) gave a robust fluorescence response over the 10 hours compared to the traditional TetR system.

Since placing the QUAS sequence 10 base pairs upstream of the T7 promoter gave the highest protein production in the presence of QF (Fig. 2b), we studied whether this configuration would significantly increase the sensitivity to aTc (Fig. 4b). To control the activation of *QF*, this circuit was designed to produce QF in the presence of aTc by placing a TetO site upstream of the *QF* gene and the expression of *GFP* was regulated by the activation of QF binding to the QUAS binding site. In the absence of aTc, the TetR proteins repress the transcription of *QF* preventing it from activating the expression of *GFP* (Fig. 4b, top circuit). In the presence of aTc, the TetR proteins no longer bind to the TetO site, enabling T7RNAP to bind to the T7 promoters and transcribe *QF* for the activation of *GFP* transcription (Fig. 4b, bottom circuit). When aTc was added to the system, an increase in background expression was observed (0 ng/ml aTc) (Fig. 4b), however, the GFP output was significantly higher in the presence of aTc compared to the traditional TetR system (Fig. 4b, dotted line and Supplementary Fig. 5).

Biological signal filters describe genetic devices that can be activated only by an input signal confined to a certain range and activation is blocked with inputs outside of that range. High-pass genetic filter devices are only activated with high input signal. We next sought to construct a high-pass filter device by coupling the benefit of the tight off observed with QUAS upstream of the T7 promoter and the higher expression level observed when *QF* was constitutively expressed. To decrease the background expression in the absence of aTc, we designed this circuit to produce more TetR proteins by removing the T7 terminator sequence between the *QF* and the *tetR* genes (Fig. 4c). The T7 terminator encodes a DNA sequence that causes *T7RNAP* to terminate transcription and is placed after each gene to prevent *T7RNAP* read-through^47^. Removing the T7 terminator after *QF* enhances the transcription of *tetR* because the T7 promoter upstream of *QF* continues to transcribe through the T7 promoter that drives the expression of the Tet repressor gene and ultimately produces more TetR proteins. These TetR proteins repress the transcription of *tetR* and *GFP*. This circuit design results in the repression of *GFP* expression to background levels. The addition of aTc removes the transcriptional repression and results in robust expression of *GFP* that resembles a high-pass filter sensor where only high concentrations of aTc activate gene expression (Fig. 4c). Notably, this high-pass filter has high expression levels in the first 3 h after inducing with aTc, almost eight times higher than that seen with the traditional TetR system (Fig. 4c, dotted line and Supplementary Fig. 5).

To explore an approach for the mass-production of mRNA and protein, we designed a circuit that constitutively expressed *QF* with QUAS located directly upstream a T7 promoter and *GFP* (Fig. 4d). A TetO site was added directly downstream of the T7 promoter driving *GFP* expression to make the expression of *GFP* inducible (Fig. 4d). In the absence of aTc, the TetR proteins bind to the TetO site, preventing T7RNAP from binding and transcribing *GFP* (Fig. 4d, top circuit). The addition of aTc de-represses the transcription of *GFP* (Fig. 4d, bottom circuit). In this design, the QF transcription factor appears to over-power the Tet repressor proteins and activates high *GFP* expression regardless of whether aTc is in the culture in the first few hours. By hour 5, aTc enables greater expression of *GFP* compared to the traditional TetR system (Fig. 4d, dotted line and Supplementary Fig. 5) for up to 9 h after induction. This circuit design is capable of producing at least thirty times higher gene expression compared to the traditional TetR system.

## Discussion

Developing unique genetic circuits and repurposing genetic regulatory elements to reprogram cellular function has led to new opportunities for clinical applications^48,49^ and has the potential to influence the therapeutic response to public health crises. Here, we demonstrate the utility of an unconventional approach in which eukaryotic genetic elements are used to regulate gene expression in bacteria. Moreover, we demonstrate that this approach is highly versatile and can be engineered to provide tight regulatory control or amplified gene expression based upon the position of the eukaryotic QUAS relative to the T7 bacteriophage promoter. Furthermore, due to its eukaryotic origins, this system is orthogonal to existing regulatory tools in bacteria and therefore can be used to generate more sophisticated genetic circuits for therapeutic and research efforts than currently available.

The eukaryotic fungus *Neurospora crassa* uses a genetic circuit to direct the catabolism of quinic acid as a carbon source in certain metabolic states. This cluster of genes is known as the Q system and utilizes genes from the *qa* gene cluster. In brief, the gene cluster is on when QF binds to QUAS and activates downstream gene expression, and it is off when the negative regulator QS associates with QF and prevents its binding to activate transcription. The natural circuit switches between states via quinic acid. Quinic acid prevents QS from binding to QF, freeing the QF activation domain and re-activating gene expression. While we found that QUAS and the QF transcription factor are functional in prokaryotes, we have not seen any effect of adding QS to the system, suggesting that this arm of the Q system is not functional in this context. One possibility is that the QS function requires post-translational modifications that do not occur in bacteria. More importantly, we have found that QUAS has distinct regulatory functions in the absence of these other components of the Q system when placed upstream of the T7 promoter in bacteria. In this location, it inhibits transcription and does so with sufficient efficacy to enable cell survival when controlling the expression of toxic genes.

While the functional consequence of placing QUAS directly upstream of the T7 promoter is clear, the molecular mechanisms by which it prevents transcription in bacteria have not been identified. We hypothesize that native bacterial repressor proteins may bind to QUAS and block T7RNAP from binding the adjacent T7 promoter. This possibility is consistent with the repressive nature of gene regulation in bacteria. This unknown repressor protein could be displaced by QF due to its higher binding affinity for QUAS, enabling T7RNAP to bind to the T7 promoter and initiate gene expression. Alternatively, QUAS may introduce conformational changes to the local DNA structure that makes the adjacent T7 promoter inaccessible to T7RNAP, and that this inhibition is released when the QUAS is bound by QF. Mechanistically this could be analogous to heterochromatin in eukaryotes, with QF functioning like a pioneer transcription factor that stabilizes the DNA structure in a configuration compatible with RNA polymerase binding and transcription initiation.

The impact of placing QUAS downstream of the T7 promoter highlights the dual function of this *cis*-regulatory element when utilized in bacteria. In this position QUAS does not have a repressive function but enables significant QF-dependent amplification of gene expression. Levels of *GFP* expression in the presence of QF showed amplification from the T7 promoter and were much greater than that of T7 alone, exceeding levels of gene expression not seen from any other bacterial expression system. One hypothesis for why the highest gene expression level is observed when QUAS is placed fifteen base pairs downstream of the promoter is that this position gives more space for the QF transcription factor and the T7RNAP to both bind and enable more robust expression of the downstream gene.

Altogether, it is important to note that QUAS, while typically described as an upstream activator, facilitates both the activation and repression of gene expression in bacteria. Moreover, we demonstrate that it can function both upstream and downstream of a promoter sequence. Based upon these observations we would suggest that it is more appropriate to refer to the QUAS sequence as a QF binding site rather than QUAS which more aptly describes transcriptional activation from an upstream *cis*-regulatory element.

Mechanistically, it is noteworthy that QF can function as a transcriptional activator in a bacterial system because bacteria rarely utilize activators for transcriptional regulation since they typically use repression and de-repression systems for regulating gene expression. Bacterial synthetic biologists have long sought a transcriptional activator to increase gene expression levels. To date, all bacterial systems, with a few exceptions, are repressive in nature. Of these exceptions, the LuxR system is an activator that turns on gene expression at *pLuxI* promoters when the molecule 3-oxy-hexanoyl-L-homoserine lactone, or autoinducer AI is present^50^. In addition, efforts have been made to achieve activation at bacterial promoters that requires the use of catalytically inactive Cas9 and guide RNAs^51^. Some success has been achieved with increasing expression of T7 promoters in bacteria, but this requires protein engineering^23^. The Q system, on the other hand, requires a single activating protein (QF) and binding domain (QUAS), which is not limited by requiring unique inducers that function to activate gene expression at T7 promoters. The influence that eukaryotic regulatory elements may have on endogenous bacterial gene expression have not been directly tested by our experimental approach; however, they seem unlikely to be significant since activators have not evolved in this context. From a synthetic biology perspective, the efficacy of QF and QUAS in bacteria has significant utility as an independent regulatory system. For example, the large range of control with respect to the orthogonal T7 promoter offers unique advantages in genetic circuit design. Other systems, such as the TetR and LacI systems, function within a narrow range downstream of the T7 promoter, with the upstream positions viable only in unique applications^52–54^. The freedom of placing QUAS relatively far from the promoter allows the placement of additional modules, thereby enabling the design of complex genetic circuits.

We demonstrate the unique programmability of the Q system in bacteria by creating genetic devices that are capable of sensing low input levels and responding with strong output signals. This has the potential to be useful in sensor and diagnostic applications. For example, synthetic biology-based diagnostics have demonstrated that programmable RNA sensors can be engineered to function in a low cost, paper-based, cell-free platform^55–61^. These diagnostics are stable at room temperature, and have the potential to meet the needs in low-resource settings. A challenge to many diagnostics is the detection and amplification of small amounts of sample to give a robust readout. Here, we demonstrate that our genetic devices are capable of maintaining a tight off state and detecting small amounts of sample to produce a readout that is significantly higher than standard devices. Coupling our system to cell-free platforms may help improve the rapid detection of a pathogen, contaminant, or antigen that needs to be detected to inform a proper response. In addition, with the emergence of mRNA-based vaccines as a therapeutic intervention, it is possible that these genetic tools can be used to produce high levels of mRNA and protein for biotechnology applications.

## Methods

### QUAS T7 promoter construction

A single 16-mer QUAS (GGGTAATCGCTTATCC) was oriented upstream or downstream of the T7 promoter sequence. A degradation tag was PCR amplified onto the C-terminal end of the *GFP* gene and cloned into the QUAS-0-T7 or T7-0-QUAS vectors by restriction enzyme cloning (Supplementary Table 1). The degradation tag sequence (DAS + 4) was chosen to leverage bacterial ssrA degradation machinery to allow for the rapid degradation of GFP and CcdB^53^. To maintain transcription in the T7-0-QUAS expression system, the two base pairs immediately downstream of the promoter were conserved (GG), and QUAS was inserted after these two base pairs (T7-0-QUAS) (Supplementary Table 2). Downstream spacing is notated by counting the number of base pairs between the conserved nucleotides (GG) at the 5′ end of T7 and the start of the QUAS. Upstream spacing plasmids (QUAS-5-T7, QUAS-10-T7, and QUAS-15-T7) and downstream spacing plasmids (T7-5-QUAS, T7-10-QUAS, and T7-15-QUAS) were cloned using gBlocks (IDT) (Supplementary Table 2). For the downstream designs, care was taken to keep the spacing of the end of QUAS and the ribosome binding site (RBS) the same, in addition to the spacing between the RBS and the start codon (Supplementary Table 2). The gBlocks were digested and ligated into backbones containing the high copy ColEI replication of origin, ampicillin resistance, and expression backbone containing *GFP* with a degradation tag. The *QF* expression plasmid was constructed by PCR amplifying the *QF2* sequence from pAC-7-QFBDAD (Addgene plasmid #46096)^31^ (Supplementary Table 1) and inserting it downstream of a T7lacO promoter in a vector backbone with kanamycin resistance and a high copy p15A origin of replication using restriction enzyme cloning. The QUAS-0-T7-*ccdB* plasmid was constructed by PCR amplifying *ccdB* with the degradation tag (Supplementary Table 1) and using restriction enzyme cloning to insert the amplicon into the QUAS-0-T7 vector in place of GFP. All plasmid transformations to build the constructs used DH5α chemically competent cells (ThermoFisher). The plasmids constructed in this study (Supplementary Table 3) have been deposited in Addgene.

### *GFP* expression experiments

*GFP* expression experiments were conducted with chemically competent BL21(DE3) *E. coli* (ThermoFisher) engineered to express *T7RNAP* when induced with isopropyl β-D-1-thiogalactopyranoside (IPTG). Expression experiments without QF were conducted by transforming the *GFP* expression plasmid alone (e.g., single transformation) into the BL21(DE3) cells. Expression experiments with *QF* were conducted by co-transforming the *GFP* expression plasmid and the *QF* expression plasmid into BL21(DE3). For each experiment, three colonies of transformed BL21(DE3) were picked and grown overnight in LB (Fisher Scientific) with antibiotic selection (100 μg/mL carbenicillin and/or 50 μg/mL kanamycin) at 37 °C and shaken at 280 RPM. The overnight culture was diluted 1:50 in LB with antibiotic selection, grown at 37 °C, and shaken at 280 RPM. This dilution started at about 0.03 OD and took ~1–2 h of growth (lag phase) before the cultures reached an OD of 0.2 (the beginning of log phase). This log phase OD was determined by obtaining growth curves for all experiential conditions (Supplementary Fig. 6). Cultures were induced with 0.5 mM IPTG at an OD_600_ (Synergy HTX Reader, Biotek) of ~0.2. Induction served as the time zero point for all experiments. This plate reader corrects the pathlength for each plate used and 300 μl of bacterial culture was consistently taken for all OD measurements.

### Flow cytometry

Samples were diluted 1:400 in PBS and analyzed on a CytoFLEX S cytometer (Beckman Coulter) using a 488 nm laser and a 525/50 filter for Figs. 2, 3, 4 and Supplemental Fig. 5. All other figures were constructed using data obtained from a DXP flow cytometer (Cytek) using a 488 nm laser and a 530/30 filter. Measurements were taken at time points 1–10 h post induction with IPTG. Populations of 10,000 cells were used to calculate GFP fluorescence. Flow cytometery data were collected using CytExpert (version 2.4.0.28 Beckman Coulter) and flow data were analyzed using Flowing Software (Cell Imaging Core, Turkey Centre for Biotech) or FlowJo and MATLAB (The MathWorks, Inc.). Bacterial cells were identified and gated by plotting the side scatter (SSC) vs. forward scatter (FSC) on a log scale (Supplementary Fig. 7). GFP fluorescence data in Figs. 1, 2, 3, and Supplemental Fig. 2 were normalized by dividing the mean fluorescence of each sample by that of the T7 control 1 h after induction.

### *CcdB* expression experiments

QUAS-0-T7 expression experiments without QF were conducted by transforming the expression plasmid alone (without QF) into BL21(DE3) cells. Experiments with QUAS-0-T7 and QF were conducted by co-transforming the expression plasmid with the *QF* expression plasmid into BL21(DE3) cells. Three colonies of transformed BL21(DE3) were picked and grown overnight in LB (Fisher Scientific) with antibiotic selection at 37 °C and shaken at 280 RPM. The overnight culture was diluted 1:50 in LB with selection antibiotics and grown at 37 °C with shaking at 280 RPM. Cultures were induced with 10 μM IPTG at an OD_600_ (Synergy HTX Reader, Biotek) of ~0.2. IPTG induction of *T7RNAP* was used as the time point zero for all experiments. For each hour (0–4) post induction, samples were serially diluted 1:10 in PBS and plated on LB agar plates with antibiotic selection. These plates were incubated at 37 °C overnight, and colonies were counted the next day to calculate colony forming units (CFUs).

### Q and TetR system

Genetic circuits containing both TetR and Q system genetic parts were synthesized as gene fragments (IDT) (Supplementary Fig. 2) and inserted into plasmid backbones. A plasmid containing both T7 TetO QF and T7 LacO TetR was PCR amplifying from the T7 LacO TetR region and inserting it into the T7 TetO QF plasmid (Supplementary Fig. 1). Constructs containing *GFP* were placed in backbones with the high copy ColE1 origin of replication and ampicillin resistance. Circuits expressing *QF* and/or *TetR* contain the high copy p15A origin of replication and kanamycin resistance. *GFP* expression was measured using a flow cytometer as described in the *GFP* expression experiments; however, cultures were induced at OD_600_ ~ 0.1. Anhydrotetracycline (cat. no. AAJ66688MA, Alfa Aesar) was dissolved in ethanol according to manufacturer’s directions and stored at −20 °C. Fresh dilutions were prepared for each experiment using water to achieve the reported concentrations. GFP expression was measured using a flow cytometer, as described in the *GFP* expression experiments.

### Growth curves

Bacterial colonies in biological triplicates were grown overnight in 1.5 mL of LB with antibiotics. The following morning, OD_600_ was measured, and the overnight cultures were diluted 1:50 in fresh LB and antibiotics. Two milliliters of diluted culture were added to 15 mL, round-bottom tubes (Falcon), one tube for every measurement. Starting at the time of dilution, OD_600_ measurements were taken every 30 min using the same procedure described in *GFP* expression experiments (Supplementary Fig. 6). A new 2-mL, culture sample was used for each measurement to maintain the same air-to-media ratio for all measurements.

### Reporting summary

Further information on research design is available in the Nature Research Reporting Summary linked to this article.

## Supplementary information

Supplemental Information

Description of Additional Supplementary Files

Supplementary Software

Reporting Summary

## Data Availability

All data collected to evaluate the conclusions in this work are presented in the paper and/or in the Supplementary Materials. All plasmids generated in this study have been deposited in Addgene (Supplementary Table 3). Reagents or additional data are available from the corresponding author upon request. Source data are provided with this paper.

## Source data

Source Data

## Code availability

All MATLAB code used to analyze the data can be found in the Supplementary Software file.
