# Supplementary material for: Enhanced regulation of prokaryotic gene expression by a eukaryotic transcriptional activator
Source: Nat Commun. 2021 Jul 5;12:4109. doi: 10.1038/s41467-021-24434-9 (PMC8257575; doi:10.1038/s41467-021-24434-9)
Supplement: Supplementary file 1 — Supplemental Information [file 41467_2021_24434_MOESM1_ESM.pdf]

Supplementary Information: Enhanced regulation of prokaryotic gene expression by a eukaryotic transcriptional activator

I. Cody MacDonald*[1], Travis R. Seamons*[1], Jonathan C. Emmons[1], Shwan B. Javdan[1], and Tara L. Deans[1]†

[1]Department of Biomedical Engineering, University of Utah, Salt Lake City, UT 84112

*ICM and TRS contributed equally to this work.

†To whom correspondence should be addressed: tara.deans@utah.edu

Supplementary Tables

Gene name	(5′) Forward Primer (3′)	(5′) Reverse Primer (3′)
QF insert	CCGGATCCAACATGCCACC	GGATCGGTTAACGCGGCC
GFP-ssrA	CCTGGCGGCAGCGCAAAAGA-TGGTGAGCAAGGGCGAGGAG	CCGTTTAGAGGCCCCAAGGGGTTATGCTAG-GATATCAAGCTTCTAAGACGCGTCCGCGTAG
ccdB	GTACATATGATTCAGTTTAAGGTTTACACC	AAGCTT TTAAGACGCGTCC
T7 LacO TetR	TCAGTGCGGCCGCCAGTTTACTTTGCAGGGC	TCAGTCGATATAAGTTGTGCGGCC

Supplementary Table 1: List of primers used for PCR.

QUAS-0-T7	QUAS T7 GFP<br>GGGTAATCGCTTATCCTAATACGACTCACTATA–––RBS–––ATG
T7-0-QUAS	T7 QUAS GFP<br>TAATACGACTCACTATAGGGGGTAATCGCTTATCC–––RBS–––ATG
T7lacO	T7 LacO GFP<br>TAATACGACTCACTATAGG GGAATTGTGAGCGGATAACAATT–––RBS–––ATG
pLacO	LacO -35 LacO -10 GFP<br>ATAAATGTGAGCGGATAACATTGACATTGTGAGCGGATAACAAGATACT–––RBS–––ATG
QUAS-0-T7TetO	QUAS T7 TetO GFP<br>GGGTAATCGCTTATCCTAATACGACTCACTATAGGTCCCTATCAGTGATAGAGA–––RBS–––ATG
QUAS-5-T7	QUAS T7 GFP<br>GGGTAATCGCTTATCCCACAT TAATACGACTCACTATAGG–––RBS–––ATG
QUAS-10-T7	QUAS T7 GFP<br>GGGTAATCGCTTATCCCTATTATCATTAATACGACTCACTATAGG–––RBS–––ATG
QUAS-15-T7	QUAS T7 GFP<br>GGGTAATCGCTTATCCCATTATTATTATCATTAATACGACTCACTATAGG–––RBS–––ATG
T7-5-QUAS	T7 QUAS GFP<br>TAATACGACTCACTATAGGGTGTAGGGTAATCGCTTATCC–––RBS–––ATG
T7-10-QUAS	T7 QUAS GFP<br>TAATACGACTCACTATAGGCTATTATCATGGGTAATCGCTTATCC–––RBS–––ATG
T7-15-QUAS	T7 QUAS GFP<br>TAATACGACTCACTATAGGCATTATTATTATCATGGGTAATCGCTTATCC–––RBS–––ATG
T7-LacO-TetR	T7 LacO<br>TAATACGACTCACTATA GGGGAATTGTGAGCGGATAACAATTCCCGACTAGAAATAATTTTGTTTAA<br>TetR<br>–––RBS––– GAGGATCCATG

Supplementary Table 2: Synthesized DNA parts for placement of QUAS relative to the T7 promoter. QUAS-0-T7 has one QUAS site (dark blue) directly upstream of the T7 promoter (orange). QUAS-0-T7TetO adds a TetO (red) site two base pairs downstream of the promoter. QUAS was moved -5, -10, and -15 base pairs upstream and downstream of the T7 promoter. T7-0-QUAS has one QUAS site two base pairs downstream of the T7 promoter. This is the control referred to as T7 in other figures. pLacO is an example of an engineered promoter that utilizes the native RNAP machinery. The -10 (Pribnow box) and -35 hexamer sites are named according to the number of base pairs upstream of the transcriptional start site. Endogenous RNAP binds to promoter sequence at the -10 and -35 sites. The two LacO sites (grey) allow for LacI binding and repression of the promoter. In each case, the ribosome binding site (blue RBS)

and GFP (green ATG) are located downstream of the promoter. The expression of the *tetR* (pink ATG) gene is regulated by the binding of LacI repressor proteins.

Plasmid	Addgene ID
T7-LacO-TetR_p15A	171664
QUAS-0-T7-TetO-GFP_T7-LacO-TetR_ColE1	171663
QUAS-0-T7-TetO-GFP_ColE1	171662
T7-LacO-QF-T7-TetO-TetR_p15A	171661
QUAS-5-T7-TetO-GFP_ColE1	171660
T7-TetO-QF_T7-LacO-TetR_p15A	171659
T7-15-QUAS-GFP_ColE1	171658
T7-10-GFP_ColE1	171657
T7-5-QUAS-GFP_ColE1	171656
QUAS-15-T7-GFP_ColE1	171655
QUAS-10-T7-GFP_ColE1	171654
QUAS-5-T7-GFP_ColE1	171653
QUAS-0-T7-ccdB_ColE1	171652
T7-LacO-QF_p15A	171651
T7-0-QUAS-GFP_ColE1	171650
QUAS-0-T7-GFP_ColE1	171649
T7-LacO-GFP_ColE1	171648

Supplementary Table 3: Plasmids used in this study. All plasmids built for this study are available for Academic and Nonprofit Institutions only from Addgene.

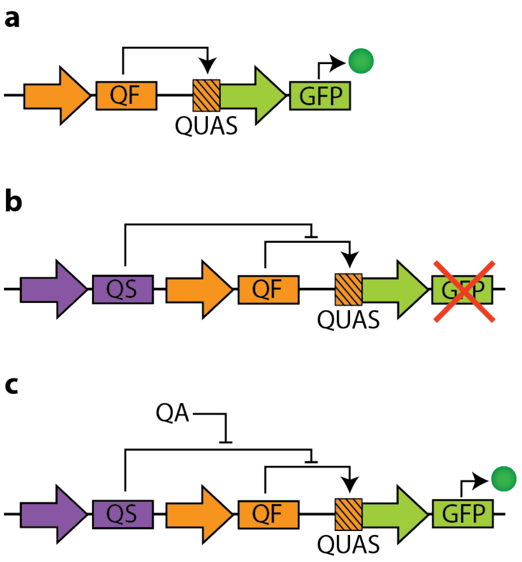

Supplementary Figure 1: Components of the Q regulatory system from *Neurospora crassa*. **a**, The transcription factor *QF* (orange) activates QUAS-regulated genes to produce downstream genes or effector proteins (here, *GFP*, green). **b**, QF activity is blocked by the binding of QS (purple) to QF. **c**, The binding of QS to QF is reversed with the addition of quinic acid (QA), enabling QF to activate the transcription of downstream genes (here, *GFP*).

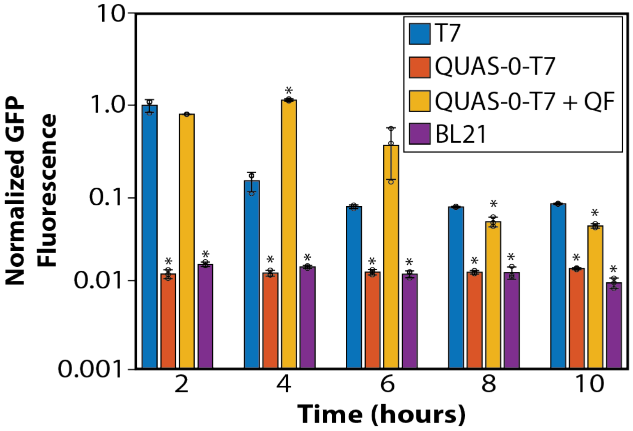

Supplementary Figure 2: T7 expression and untransformed BL21 (DE3) cell lines. *GFP* expression from T7 (blue), QUAS-0-T7 (orange), QUAS-0-T7 +QF (yellow), and untransformed BL21 (purple) cells over 10 hours. Fluorescence values were normalized to the T7 control at 2 hours after adding 0.5mM IPTG, and plotted on a log scale. Each experiment consisted of generating data from at least three separate bacterial colonies grown in overnight cultures, where circles represent individual data points in the plots. These experiments were repeated independently at least three times with similar results. The geometric mean of each sample was calculated via FlowJo, and error bars indicate standard deviation. A two-tailed t-test was performed to determine statistical significance ($P<0.004$) between the T7 control and components of the Q system with QUAS placed 5 base pairs downstream of the T7 promoter. An aster (*) represents statistical significance. The error bars indicate 95% confidence intervals of the mean of fluorescence, and data are presented as mean ± standard deviation.

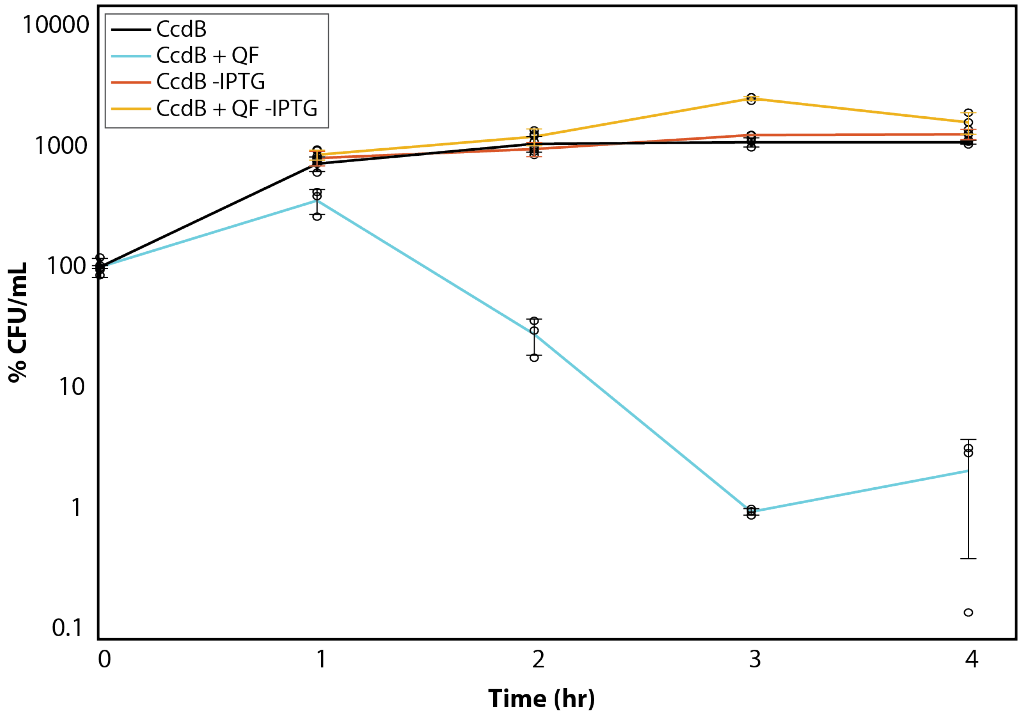

Supplementary Figure 3: ccdB induced death including uninduced controls. QUAS-0-T7 driving *ccdB* expression without IPTG (orange line), QUAS-0-T7 driving *ccdB* expression induced with 10uM IPTG (black line), QUAS-0-T7 driving *ccdB* co-transformed with a plasmid constitutively expressing QF without IPTG (yellow line), and QUAS-0-T7 driving *ccdB* co-transformed with QF expression plasmid induced with 10uM IPTG (blue line). Each experiment consisted of generating data from at least three separate bacterial colonies grown in overnight cultures, where circles represent individual data points in the plots. These experiments were repeated independently at least three times with similar results. The geometric mean of each sample was calculated via FlowJo, and error bars indicate standard deviation. The error bars indicate 95% confidence intervals of the mean of fluorescence, and data are presented as mean ± standard deviation.

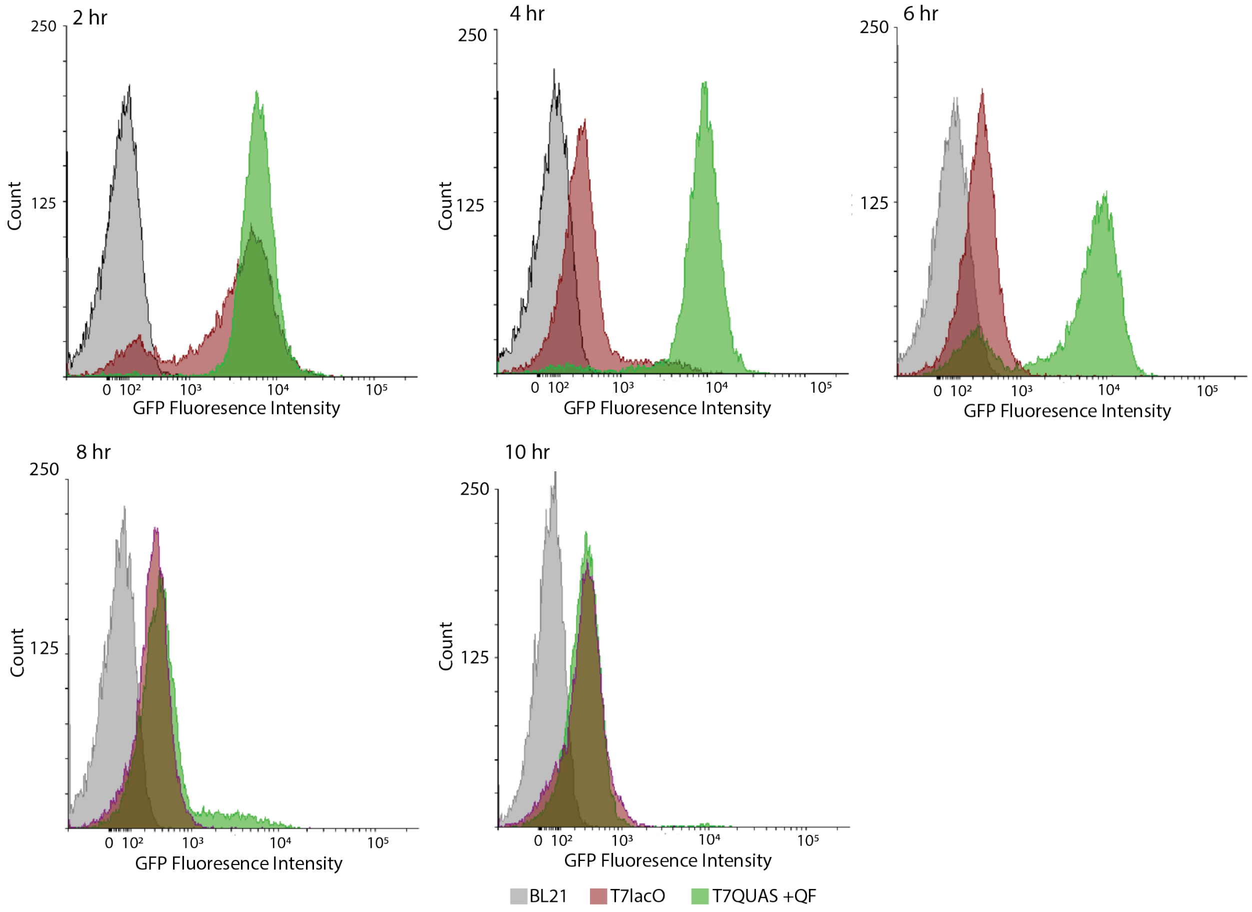

Supplementary Figure 4: Overlay histograms of GFP fluorescence from T7 promoters. Flow cytometry histograms include BL21 (grey) as an untransformed control without exogeneous DNA, T7 constitutively expressing *GFP* (pink) and T7-0-QUAS +QF (green). Hours represent time post IPTG induction of *T7RNAP*.

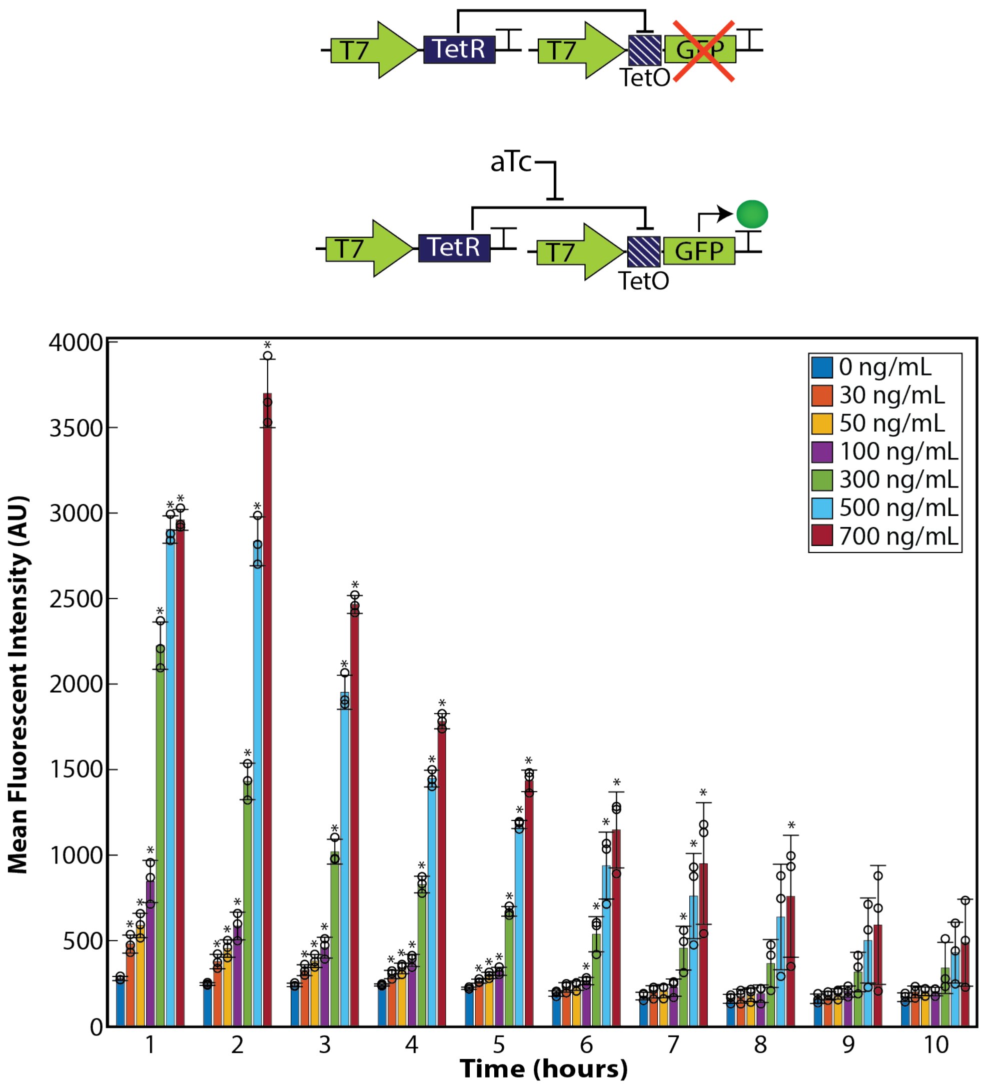

Supplementary Figure 5: Traditional TetR system. Schematic in the absence of aTc (top). The TetR proteins bind to the TetO site and prevent T7RNAP from binding. Schematic of the presence of aTc, which removes the TetR proteins from operator sites, allowing T7RNAP to bind and transcribe *GFP* (bottom). Flow cytometry quantifying GFP fluorescence at various aTc concentrations for all constructs using flow cytometry over a 10 hour period. The induction of 0.5 mM IPTG initiates the transcription of *T7RNAP* (added at time zero). Each experiment consisted of generating data from at least three separate bacterial colonies grown in overnight cultures, where circles represent individual data points in the plots. These experiments were repeated independently at least three times with similar results. The geometric mean of each

sample was calculated via FlowJo, and error bars indicate standard deviation. A two-tailed t-test was performed to determine statistical significance ($P<0.05$) between the uninduced control with increasing concentrations of aTc. The error bars indicate 95% confidence intervals of the mean of fluorescence, and data are presented as mean ± standard deviation. An aster (*) represents statistical significance.

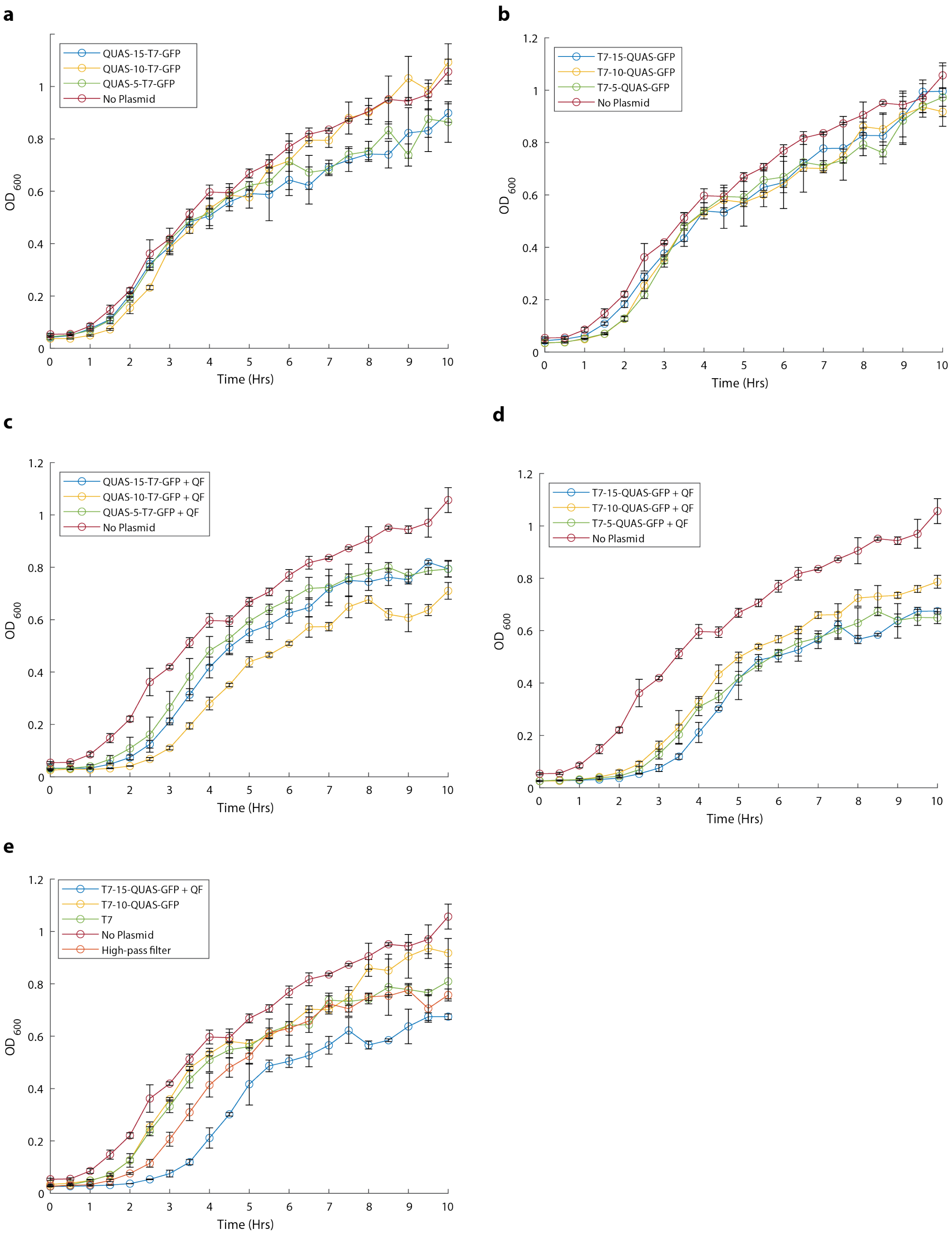
a
QUAS-15-T7-GFP
QUAS-10-T7-GFP
QUAS-5-T7-GFP
No Plasmid
OD 600
Time (Hrs)
b
T7-15-QUAS-GFP
T7-10-QUAS-GFP
T7-5-QUAS-GFP
No Plasmid
OD 600
Time (Hrs)
c
QUAS-15-T7-GFP + QF
QUAS-10-T7-GFP + QF
QUAS-5-T7-GFP + QF
No Plasmid
OD 600
Time (Hrs)
d
T7-15-QUAS-GFP + QF
T7-10-QUAS-GFP + QF
T7-5-QUAS-GFP + QF
No Plasmid
OD 600
Time (Hrs)
e
T7-15-QUAS-GFP + QF
T7-10-QUAS-GFP
T7
No Plasmid
High-pass filter
OD 600
Time (Hrs)

Supplementary Figure 6: Growth curves of BL21 *E. coli*. a Growth curves of bacteria transformed with plasmids containing QUAS upstream at -15 (blue), -10 (yellow), or -5 (green) nucleotides from the T7 promoter. No plasmid control (red) is BL21(D3) not transformed with any plasmids. **b**, Growth curves of bacteria transformed with plasmids containing QUAS downstream at +15 (blue), +10 (yellow), or +5 (green) nucleotides from the T7 promoter. No plasmid control (red) is BL21(D3) not transformed with any plasmids. **c**, Growth curves of bacteria co-transformed with plasmids containing QUAS upstream at -15 +QF (blue), -10 +QF (yellow), or -5 +QF (green) nucleotides from the T7 promoter. No plasmid control (red) is bacteria not transformed with any plasmids. **d**, Growth curves of bacteria transformed with plasmids containing QUAS downstream at +15 +QF (blue), +10 +QF (yellow), or +5 +QF (green) nucleotides from the T7 promoter. No plasmid control (red) is bacteria not transformed with any plasmids. **e,** Growth curves of bacteria transformed with the high-pass filter (orange) and compared to a T7-GFP control (green), and QUAS +10 nucleotides (yellow) and +15 nucleotides +QF (blue). Each experiment consisted of generating data from at least three separate bacterial colonies grown in overnight cultures, where circles represent individual data points in the plots. These experiments were repeated independently at least three times with similar results. The geometric mean of each sample was calculated via FlowJo, and error bars indicate standard deviation. The error bars indicate 95% confidence intervals of the mean of fluorescence, and data are presented as mean ± standard deviation.

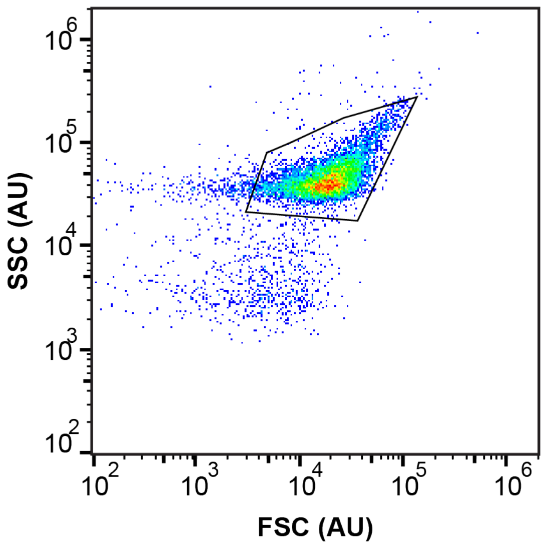

Supplementary Figure 7: Gating strategy. Bacteria populations were identified by plotting side scatter (SSC) and forward scatter (FSC) on a log scale. The gated population (in outlined area) was used for all subsequent measurements of fluorescence.
