# Supplementary material for: Enhanced regulation of prokaryotic gene expression by a eukaryotic transcriptional activator
Source: Nat Commun. 2021 Jul 5;12:4109. doi: 10.1038/s41467-021-24434-9 (PMC8257575; doi:10.1038/s41467-021-24434-9)
Supplement: Supplementary file 2 — Description of Additional Supplementary Files [file 41467_2021_24434_MOESM2_ESM.pdf]

Title: Supplementary Software

Description: Matlab code and functions for generating all of the figures in the manuscript.
