# Supplementary material for: Enhanced regulation of prokaryotic gene expression by a eukaryotic transcriptional activator
Source: Nat Commun. 2021 Jul 5;12:4109. doi: 10.1038/s41467-021-24434-9 (PMC8257575; doi:10.1038/s41467-021-24434-9)
Supplement: Supplementary file 3 — Supplementary Software [file 41467_2021_24434_MOESM3_ESM.pdf]

Supplementary Software File: Enhanced regulation of prokaryotic gene expression by a eukaryotic transcriptional activator

I. Cody MacDonald*[1], Travis R. Seamons*[1], Jonathan C. Emmons[1], Shwan B. Javdan[1], and Tara L. Deans[1]†
[1]Department of Biomedical Engineering, University of Utah, Salt Lake City, UT 84112
[*]ICM and TRS contributed equally to this work.
†To whom correspondence should be addressed: tara.deans@utah.edu

```
%% Figure 1a
%QUAS-0-T7-GFP
clear

%means of fluorescence (AU)
data = [56.96129566 60.44626743 63.20010091 56.24219216
62.35102722 64.2386558  60.27753439 52.75117132
59.40496299 70.54991628 63.72283058 55.69958971
1079.090791 3361.475444 4520.952936 4143.139863
983.1715876 3331.824232 4399.426639 4950.997216
959.1776761 3239.324381 4532.006766 5126.431373];

t = 1:4;

%User-defined Function ("Functions for Plotting") must be downloaded first
QplotDxP(t, data, '0 Base Pairs Upstream', 'QUAS(0)T7', 'QUAS(0)T7 + QF');

%Test for Significance
T7LacO = [3439  3687    1960    1007    625 459 381 376 383 390
3584    4096    2081    887 542 416 382 374 376 382
3649    3547    1570    703 430 381 381 375 385 402

4167    4215    1775    1014    742 493 478 461 498 522
4483    4738    2064    742 491 439 458 470 480 526
4014    3668    1301    550 415 382 404 443 490 530

3581    2898    1220    632 537 605 491 454 472 504
3453    2656    860 443 318 382 398 443 469 514
3221    2198    719 414 319 372 399 422 462 507];

norm = mean(T7LacO(:,1)); % Divide results by this value to normalize data
hours = t(end); %total number of hours for the experiment

%data normalization
data = data./norm;
I = data(1:3, :);
II = data(4:6, :);
III = T7LacO(:, 1:hours);

[h_I_II, pvalue_I_II] = ttest2(I, II);
[h_I_III,pvalue_I_III] = ttest2(I, III);
[h_II_III,pvalue_II_III] = ttest2(II, III);

% h is the test result assuming a significance level of 0.05(*).
% h = 1 if the results are statistically significant, 0 if they
```

```
% not.
% pvalue is the pvalue for the statistic.

%% Figure 1b
%T7-0-QUAS-GFP
clear

%means of fluorescence (AU)
data = [3070.719687 4482.764417 2795.686774 763.9140835
3102.055799 4950.786733 3430.118685 988.5410147
3069.771998 4878.975137 3379.298036 1059.930775
3188.00839  5761.677368 7552.094863 9476.767994
3242.393468 5849.58325  7637.11314  9542.870428
3463.47571  5885.640451 7693.600658 9330.85221];

t = 1:4;

%User-defined Function ("Functions for Plotting") must be downloaded first
QplotDxP(t, data, '0 Base Pairs Downstream', 'T7(0)QUAS', 'T7(0)QUAS + QF');

%statistics
%data normalization
T7LacO = [3439  3687    1960    1007    625 459 381 376 383 390
3584    4096    2081    887 542 416 382 374 376 382
3649    3547    1570    703 430 381 381 375 385 402

4167    4215    1775    1014    742 493 478 461 498 522
4483    4738    2064    742 491 439 458 470 480 526
4014    3668    1301    550 415 382 404 443 490 530

3581    2898    1220    632 537 605 491 454 472 504
3453    2656    860 443 318 382 398 443 469 514
3221    2198    719 414 319 372 399 422 462 507];

norm = mean(T7LacO(:,1)); % Divide results by this value to normalize data
data = data./norm;
hours = t(end); %total number of hours for the experiment

I = data(1:3, :);
II = data(4:6, :);
III = T7LacO(:, 1:hours);

[h_I_II, pvalue_I_II] = ttest2(I, II);
[h_I_III,pvalue_I_III] = ttest2(I, III);
[h_II_III,pvalue_II_III] = ttest2(II, III);

%% Figure 1c
%CcdB Experiment
clear

%Figure 1c
%each column is a new timepoint
data = [0.946428571 7.678571429 9.107142857 9.464285714 10.71428571
0.857142857 7.321428571 11.78571429 10.53571429 10
```

```
1.196428571 5.892857143 9.464285714 11.25   10.53571429
1.03030303  3.787878788 0.363636364 0.009393939 0.001515152
0.984848485 2.575757576 0.181818182 0.010151515 0.03030303
0.984848485 4.090909091 0.303030303 0.010606061 0.033333333];
data = data.*100; %convert to %CFU/mL

ccdb = data(1:3, :);
ccdbQF = data(4:6, :);

%mean
b1 = mean(ccdb);
b2 = mean(ccdbQF);

%standard deviation
e1 = std(ccdb);
e2 = std(ccdbQF);

t = 0:4; %hrs

time_points1 = [];

for r = 1:length(t)
    temp = ones(1,3).*t(r);
    time_points1 = [time_points1, temp];
end

time_points2 = [];
for r = 1:length(t)
    temp = ones(1,3).*t(r);
    time_points2 = [time_points2, temp];
end

data_points = data(:)';

figure
hold on
plot(t, b1, 'k')
plot(t, b2, 'c')
plot(time_points1, ccdb(:), '.k')
plot(time_points2, ccdbQF(:), '.c')
ylim([0.1 1e4])
set(gca, 'YScale', 'log')
yticks([0.1, 1, 10, 100, 1e3, 1e4])
yticklabels({'0.1', '1', '10', '100', '1000', '10000'})
xticks([0:4])
legend('QUAS-0-T7-CcdB', 'QUAS-0-T7-CcdB + QF')
title('Figure 1g')
%errorbars
eb1 = errorbar(t, b1, e1);
eb1.Color = zeros(1,3);
eb.LineStyle = 'none';
set(get(get(eb1, 'Annotation'), 'LegendInformation'), 'IconDisplayStyle', 'off');

eb2 = errorbar(t, b2, e2);
```

```
eb2.Color = 'c';
eb2.LineStyle = 'none';
set(get(get(eb2, 'Annotation'), 'LegendInformation'), 'IconDisplayStyle', 'off');
```

Supplementary Software Figure 1: Matlab code for generating all of the plots in Figure 1: The Q system in bacteria.

```
%Code to generate graphs for Figure 2

%% Figure 2a
%% -5
%QUAS-5-T7
clear

data_5 = [112   87.5    99.5    112 108 113 99.6    105 94.6    105
92.6    90.9    96.7    109 102 105 102 95.2    92.3    105
107 116 119 109 96.9    105 90.4    87.1    92.6    93.2

1708    3676    4706    4412    2902    1358    525 333 251 234
1618    3457    3881    3270    1817    751 358 260 225 215
1935    4058    5064    4507    3390    1765    658 386 290 242

3439    3687    1960    1007    625 459 381 376 383 390
3584    4096    2081    887 542 416 382 374 376 382
3649    3547    1570    703 430 381 381 375 385 402];
t = 1:10;

%User-defined Functions ("Functions for Plotting") must be downloaded first
graph = QplotDxP(t, data_5, '5 Base Pairs Upstream', 'QUAS5T7', 'QUAS5T7 + QF');
ylim([0, 2.1]);
width = 265;
set(gcf, 'units', 'points', 'position', [10, 10, width, 0.75*width]);

%statistics
T7LacO = [3439  3687    1960    1007    625 459 381 376 383 390
3584    4096    2081    887 542 416 382 374 376 382
3649    3547    1570    703 430 381 381 375 385 402

4167    4215    1775    1014    742 493 478 461 498 522
4483    4738    2064    742 491 439 458 470 480 526
4014    3668    1301    550 415 382 404 443 490 530

3581    2898    1220    632 537 605 491 454 472 504
3453    2656    860 443 318 382 398 443 469 514
3221    2198    719 414 319 372 399 422 462 507];

hours = t(end); %total number of hours for the experiment
raw_data = data_5;
I = raw_data(1:3, :);
II = raw_data(4:6, :);
III = T7LacO(:, 1:hours);

[h_I_II, pvalue_I_II] = ttest2(I, II);
[h_I_III,pvalue_I_III] = ttest2(I, III);
[h_II_III,pvalue_II_III] = ttest2(II, III);
```

```
%% Figure 2b
% QUAS-10-T7
clear

data_10 = [77.6 72.6    84.6    86.5    103 96.7    89.7    84.9    88.4    86.1
95.7    77.3    87.2    102 100 99.2    90.2    91.9    82.4    90
82.1    70  93.1    102 102 92.8    93.9    83.6    86.3    88

2108    5039    6285    6671    5961    4247    2046    759 426 305
2185    5311    6766    7468    6801    4618    1977    707 386 311
2257    5298    6302    7354    6594    3925    1549    632 373 318

4167    4215    1775    1014    742 493 478 461 498 522
4483    4738    2064    742 491 439 458 470 480 526
4014    3668    1301    550 415 382 404 443 490 530];
t = 1:10;

%User-defined Functions ("Functions for Plotting") must be downloaded first
graph = QplotDxP(t, data_10, '10 Base Pairs Upstream', 'QUAS10T7', 'QUAS10T7 + QF');
ylim([0, 2.1]);
width = 265;
set(gcf, 'units', 'points', 'position', [10, 10, width, 0.75*width]);

%statistics
T7LacO = [3439  3687    1960    1007    625 459 381 376 383 390
3584    4096    2081    887 542 416 382 374 376 382
3649    3547    1570    703 430 381 381 375 385 402

4167    4215    1775    1014    742 493 478 461 498 522
4483    4738    2064    742 491 439 458 470 480 526
4014    3668    1301    550 415 382 404 443 490 530

3581    2898    1220    632 537 605 491 454 472 504
3453    2656    860 443 318 382 398 443 469 514
3221    2198    719 414 319 372 399 422 462 507];

hours = t(end); %total number of hours for the experiment
raw_data = data_10;
I = raw_data(1:3, :);
II = raw_data(4:6, :);
III = T7LacO(:, 1:hours);

[h_I_II, pvalue_I_II] = ttest2(I, II);
[h_I_III,pvalue_I_III] = ttest2(I, III);
[h_II_III,pvalue_II_III] = ttest2(II, III);

%% Figure 2c
%QUAS-15-T7
clear

data_15 = [89.2 92.7    97.5    97.3    108 106 106 92.5    88.5    93.3
86.8    86.8    93.7    96.7    104 106 98.2    91  93.7    101
97.8    88.3    95.4    100 105 109 104 87.3    93.9    104
```

```
1965	4186	5098	5155	4057	2164	845 403 290 262
1955	4112	4808	4581	3575	1792	711 379 280 252
2184	4689	5832	6043	5131	3278	1569	619 341 278

3581	2898	1220	632 537 605 491 454 472 504
3453	2656	860 443 318 382 398 443 469 514
3221	2198	719 414 319 372 399 422 462 507];
t = 1:10;

%User-defined Functions ("Functions for Plotting") must be downloaded first
graph = QplotDxP(t, data_15, '15 Base Pairs Upstream', 'QUAS15T7', 'QUAS15T7 + QF');
ylim([0, 2.1]);
width = 265;
set(gcf, 'units', 'points', 'position', [10, 10, width, 0.75*width]);

%statistics
T7LacO = [3439  3687	1960	1007	625 459 381 376 383 390
3584	4096	2081	887 542 416 382 374 376 382
3649	3547	1570	703 430 381 381 375 385 402

4167	4215	1775	1014	742 493 478 461 498 522
4483	4738	2064	742 491 439 458 470 480 526
4014	3668	1301	550 415 382 404 443 490 530

3581	2898	1220	632 537 605 491 454 472 504
3453	2656	860 443 318 382 398 443 469 514
3221	2198	719 414 319 372 399 422 462 507];

hours = t(end); %total number of hours for the experiment
raw_data = data_15;
I = raw_data(1:3, :);
II = raw_data(4:6, :);
III = T7LacO(:, 1:hours);

[h_I_II, pvalue_I_II] = ttest2(I, II);
[h_I_III,pvalue_I_III] = ttest2(I, III);
[h_II_III,pvalue_II_III] = ttest2(II, III);
```

Supplementary Software Figure 2: Matlab code for generating all of the plots in Figure 2: QUAS spacing upstream of the T7 promoter.

```
%Code to generate graphs for Figure 3

%% Figure 3a
%T7-5-QUAS
clear

data = [2126	4503	5291	9362	4421	4828	4184	3374	2463	1735
2525	3895	5204	5406	4629	4246	4124	2922	2458	1914
2399	4095	4301	5421	5254	4499	4824	3401	2580	1727
5728	7134	9681	4826	6350	2371	1141	720 817 591
6015	10689	12632	8990	8295	4950	2252	1024	670 584
5914	8760	11542	10629	6877	3782	1515	758 591 534];

t = 1:10;

%User-defined Functions ("Functions for Plotting") must be downloaded first
graph5 = QplotDxP_new(t, data, '5 Base Pairs Downstream', 'T7-5-QUAS', 'T7-5-QUAS + QF');
ylim([0, 8]);
width = 265;
set(gcf, 'units', 'points', 'position', [10, 10, width, 0.75*width]);

%statistics
T7LacO = [8739  12853	7568	4576	1471	963 748 1192	651 666
8488	12870	7102	2968	1615	1101	866 856 825 848
8901	12194	6330	2478	1373	835 625 625 583 529

1490	10843	6400	2840	1385	1007	771 656 663 588
2343	7954	3941	1402	746 599 536 456 433 404
2125	9674	4945	2207	1259	1034	954 967 956 882];

hours = t(end); %total number of hours for the experiment
raw_data = data;
I = raw_data(1:3, :);
II = raw_data(4:6, :);
III = T7LacO(:, 1:hours);

[h_I_II, pvalue_I_II] = ttest2(I, II);
[h_I_III,pvalue_I_III] = ttest2(I, III);
[h_II_III,pvalue_II_III] = ttest2(II, III);

%% Figure 3b
%T7-10-QUAS
clear
data = [10348	21166	20200	16104	13207	10137	7283	5166	3485	2361
10276	21627	20424	16763	12969	10359	7698	5254	3633	2263
11296	22667	23848	22126	21537	19118	17139	14012	12228	10041
10902	21719	25878	27961	27936	24025	18639	12442	7975	6295
10953	21391	26052	27507	27404	23942	12469	11195	7047	4754
11001	22426	27171	29353	29676	26810	19409	11444	6943	4274];

t = 1:10;

%User-defined Functions ("Functions for Plotting") must be downloaded first
graph10 = QplotDxP_new(t, data, '10 Base Pairs Downstream', 'T7-10-QUAS', 'T7-10-QUAS + QF');
```

```
ylim([0, 8]);
width = 265;
set(gcf, 'units', 'points', 'position', [10, 10, width, 0.75*width]);

%statistics
T7LacO = [8739  12853   7568    4576    1471    963 748 1192    651 666
8488    12870   7102    2968    1615    1101    866 856 825 848
8901    12194   6330    2478    1373    835 625 625 583 529

1490    10843   6400    2840    1385    1007    771 656 663 588
2343    7954    3941    1402    746 599 536 456 433 404
2125    9674    4945    2207    1259    1034    954 967 956 882];

hours = t(end); %total number of hours for the experiment
raw_data = data;
I = raw_data(1:3, :);
II = raw_data(4:6, :);
III = T7LacO(:, 1:hours);

[h_I_II, pvalue_I_II] = ttest2(I, II);
[h_I_III,pvalue_I_III] = ttest2(I, III);
[h_II_III,pvalue_II_III] = ttest2(II, III);

%% Figure 3c
%T7-15-QUAS
clear

data = [17871   24673   17109   11937   6289    2874    1761    1121    1012    988
15824   22774   22309   15279   9008    4224    2289    1458    1316    1367
16656   28743   19300   13000   8038    4111    2647    1605    1282    1118
26504   62114   91099   90167   83861   65143   41650   24844   10755   4907
28003   59008   84688   90913   86202   72806   48188   27354   13447   6435
25112   60802   89674   86125   81625   59635   42860   20291   8977    4852];

t = 1:10;

%User-defined Functions ("Functions for Plotting") must be downloaded first
graph15 = Qplotcyt(t, data, '15 Base Pairs Downstream', 'T7-15-QUAS', 'T7-15-QUAS + QF');
ylim([0, 8]);
width = 265;
set(gcf, 'units', 'points', 'position', [10, 10, width, 0.75*width]);
% saveas(graph15,'C:\Users\meeko\Documents\Deans
Lab\Biosensor\Writing_Biosensor\Figures\Plus15', 'epsc');

%Statistics
T7LacO = [11295 24175   14078   4671    2056    1248    895 774 679 669
19170   19952   13918   5519    2750    1668    1347    1150    963 983
7428    20767   9792    4340    2256    1547    1338    1085    1085    913]; %T7-LacO-GFP data from
CytoFLEX cytometer

hours = t(end); %total number of hours for the experiment
raw_data = data;
I = raw_data(1:3, :);
II = raw_data(4:6, :);
```

```
III = T7LacO(:, 1:hours);

[h_I_II, pvalue_I_II] = ttest2(I, II);
[h_I_III,pvalue_I_III] = ttest2(I, III);
[h_II_III,pvalue_II_III] = ttest2(II, III);
```

Supplementary Software Figure 3: Matlab code for generating all of the plots in Figure 3: QUAS spacing downstream of the T7 promoter.

```
%Code to generate graphs for Figure 4

%% Figure 4a
% QUAS(-5)T7-TetO-GFP & T7-TetO-QF_T7-LacO-TetR
clear

% Each column is a new timepoint
data = [276 279 303 324 311 326 323 340 348 422
253 249 247 264 261 248 257 237 226 251
260 261 276 290 284 279 269 264 263 246
555 483 449 413 375 378 355 355 354 381
526 456 387 367 336 301 288 254 235 235
537 478 440 409 371 341 315 301 268 250
669 565 492 454 412 375 353 368 367 399
665 584 511 470 417 379 345 291 253 228
704 605 536 489 439 390 348 311 286 251
1090	902 775 705 622 559 516 465 486 505
1013	884 757 672 595 539 461 372 319 250
1023	882 778 689 598 543 452 390 323 275
2621	2815	2202	1783	1407	1212	1051	948 808 765
2652	2587	2124	1764	1427	1209	1013	840 648 495
2767	2809	2239	1919	1530	1351	1034	778 556 390
2640	3874	3002	2392	1999	1807	1493	1343	1239	1123
2662	4209	3331	2760	2285	1964	1468	1284	1165	778
2896	4198	3328	2712	2190	1931	1395	1057	734 551
3014	3918	3806	2968	2441	2107	1781	1596	1447	1322
2812	4252	4427	3495	2934	2503	2107	1751	1626	1207
2988	4209	4749	3863	3037	2594	2053	1796	1307	820];

% means of triplicates
data_means = [263	263 275.3333333 292.6666667 285.3333333 284.3333333 283 280.3333333 279
306.3333333
539.3333333 472.3333333 425.3333333 396.3333333 360.6666667 340 319.3333333 303.3333333
285.6666667 288.6666667
679.3333333 584.6666667 513 471 422.6666667 381.3333333 348.6666667 323.3333333 302
292.6666667
1042	889.3333333 770 688.6666667 605 547 476.3333333 409 376 343.3333333
2680	2737	2188.333333 1822	1454.666667 1257.333333 1032.666667 855.3333333
670.6666667 550
2732.666667 4093.666667 3220.333333 2621.333333 2158	1900.666667 1452	1228	1046
817.3333333
2938	4126.333333 4327.333333 3442	2804	2401.333333 1980.333333 1714.333333 1460
1116.333333
226.3333333 174.3333333 347.3333333 291.6666667 218 148.6666667 140.3333333 144.3333333
129 120];

% standard deviation
error = [11.78982612	15.09966887 28.00595175 30.08875759 25.02665246 39.27255191
35.15679166 53.40724046 62.55397669 100.2014637
14.6401275  14.36430762 33.50124376 25.4820198  21.45538006 38.50973903 33.70954365
50.54041287 61.43560314 80.31396724
21.45538006 20.0083316  22.06807649 17.52141547 14.36430762 7.767453465 4.041451884
39.95414038 58.6600375  92.80265801
```

```
41.86884283 11.01514109 11.35781669 16.50252506 14.79864859 10.58300524 34.64582707
49.32544982 95.28378666 140.5643388
76.92203845 129.938447  58.70547959 84.53993139 66.0025252  81.13158037 19.03505538
86.03100216 127.5199331 193.4554212
141.8778818 190.3164032 189.0881629 200.0533262 145.6605643 82.77882177 50.92150823 151
272.7214696 288.0214112
109.8908549 181.6984682 479.3353037 449.847752  318.5576871 258.9292053 174.7264529
104.9206049 159.8968417 262.9949302
10.26320288 4.725815626 3.785938897 5.507570547 5.196152423 2.516611478 2.309401077
3.214550254 2.645751311 3];

b1 = data_means(1,:);
b2 = data_means(2,:);
b3 = data_means(3,:);
b4 = data_means(4,:);
b5 = data_means(5,:);
b6 = data_means(6,:);
b7 = data_means(7,:);
b8 = data_means(8,:);

bg = [b1(:), b2(:), b3(:), b4(:), b5(:), b6(:), b7(:)];
t = 1:10;

time_points = [];
for hour = 1:length(t)
    time_temp = [];
    for sample = 1:7
        temp = ones(1,3);
        multiplier = hour - 0.345 + (sample-1)*0.115;
        temp = temp.* multiplier;
        time_temp = [time_temp, temp];
    end
    time_points = [time_points, time_temp];
end

data_points = data(:)';

figure
hold on
bar(t, bg, 'grouped')
plot(time_points, data_points, '.k')
title({'QUAS-5-T7-TetO-GFP & T7-TetO-QF\_T7-LacO-TetR', '10-2-2020'})
xlabel('Time (Hr)')
ylabel('Mean Fluorescent Intensity (AU)')
h = legend('0', '30', '50', '100', '300', '500', '700');
title(h, 'aTc (ng/mL)')

for r = 1:7
    eb = errorbar(t-0.345 + (r-1)*0.115, bg(:,r), error(r,:));
    eb.Color = zeros(1,3);
    eb.LineStyle = 'none';
    set(get(get(eb, 'Annotation'), 'LegendInformation'), 'IconDisplayStyle', 'off');
end
```

```
%% Figure 4b
% QUAS(-10)T7-GFP & T7-TetO-QF_T7-LacO-TetR
clear

% Each column is a new timepoint
data = [1080	2425	2803	2526	2323	2171	1980	1901	1511	1213
938	2062	2370	2312	2236	2240	1884	1918	1541	1326
842	1569	1876	1822	1827	1810	1568	1349	1122	968
994	1913	2170	2126	2179	1896	1861	1769	1493	1120
926	1480	1664	1721	1815	1845	1529	1536	1373	1157
825	1355	1706	1797	1815	1700	1498	1192	1037	894
899	1343	1726	1824	1854	1656	1591	1514	1429	1207
964	1500	1926	2009	2088	1947	1935	1797	1543	1087
916	1437	1840	2036	1835	1782	1292	1136	938	761
1093	1448	1713	1807	1792	1740	1598	1428	1478	1197
1167	1565	1900	2122	2022	1834	1848	1669	1516	1083
1107	1661	1853	1817	1716	1656	1220	1159	888	706
2081	2368	2359	2352	2239	2055	1931	1695	1615	1416
2133	2634	2837	2791	2550	2406	2130	2002	1656	1322
2093	2505	2728	2529	2192	2168	1604	1360	1248	985
2166	3347	3520	3310	2999	2776	2523	2287	2048	1967
2437	3555	4122	3660	3235	3283	2725	2430	1948	1692
2332	3463	4053	3725	3457	3112	2423	2292	1823	1337
3376	4311	4824	5018	4861	4355	3806	3347	3001	2935
3322	4419	5303	5597	5570	5114	4415	4033	3690	3546
3845	4320	5192	5416	5384	4786	3977	3537	3208	3118];

% means of triplicates
data_means = [953.3333333	2018.666667	2349.666667	2220	2128.666667	2073.666667	1810.666667	1722.666667	1391.333333	1169
915	1582.666667	1846.666667	1881.333333	1936.333333	1813.666667	1629.333333	1499	1301	1057
926.3333333	1426.666667	1830.666667	1956.333333	1925.666667	1795	1606	1482.333333	1303.333333	1018.333333
1122.333333	1558	1822	1915.333333	1843.333333	1743.333333	1555.333333	1418.666667	1294	995.3333333
2102.333333	2502.333333	2641.333333	2557.333333	2327	2209.666667	1888.333333	1685.666667	1506.333333	1241
2311.666667	3455	3898.333333	3565	3230.333333	3057	2557	2336.333333	1939.666667	1665.333333
3514.333333	4350	5106.333333	5343.666667	5271.666667	4751.666667	4066	3639	3299.666667	3199.666667
212.6666667	176.3333333	348.6666667	278.3333333	211	181	167	139	141.3333333	147];

% standard deviation
error = [119.7386042	429.6420991	463.8343814	360.9044195	264.847755	230.9336124	215.5674682	323.7164397	233.7313272	183.0109286
85.03528679	292.8247485	280.8012346	215.26805	210.155498	101.6874296	201.2270691	290.2740085	236.3725872	142.3692383
33.70954365	79.00843837	100.3261348	115.3964182	140.9054056	145.934917	321.7623347	331.6358445	321.4814665	230.7928364
39.31072797	106.6723957	97.27795228	179.0484106	159.3277544	89.04680417	316.1666227	255.1280724	352.119298	256.971464
```

```
27.22743714 133.0200486 250.5081502 220.8672301 194.5481945 179.1712403 265.5830065
321.1017492 224.6604846 226.6296538
136.6394282 104.2305138 329.4576351 223.2151429 229.0356595 257.9360386 153.8440769
81.15622794 112.7312438 315.8454263
287.635765  59.92495307 250.7276078 296.1998199 367.6062205 380.6630181 314.1034861
354.1920383 353.5284053 313.5798676
8.326663998 3.511884584 12.05542755 8.326663998 4.358898944 5.291502622 8.544003745 2
7.571877794 2];

b1 = data_means(1,:);
b2 = data_means(2,:);
b3 = data_means(3,:);
b4 = data_means(4,:);
b5 = data_means(5,:);
b6 = data_means(6,:);
b7 = data_means(7,:);
b8 = data_means(8,:);

bg = [b1(:), b2(:), b3(:), b4(:), b5(:), b6(:), b7(:)];
t = 1:10;

time_points = [];
for hour = 1:length(t)
    time_temp = [];
    for sample = 1:7
        temp = ones(1,3);
        multiplier = hour - 0.345 + (sample-1)*0.115;
        temp = temp.* multiplier;
        time_temp = [time_temp, temp];
    end
    time_points = [time_points, time_temp];
end

data_points = data(:)';

figure
hold on
bar(t, bg, 'grouped')
plot(time_points, data_points, '.k')
title('QUAS(-10)T7-GFP & T7-TetO-QF\_T7-LacO-TetR')
xlabel('Time (Hr)')
ylabel('Mean Fluorescent Intensity (AU)')
h = legend('0', '30', '50', '100', '300', '500', '700');
title(h, 'aTc (ng/mL)')

for r = 1:7
    eb = errorbar(t-0.345 + (r-1)*0.115, bg(:,r), error(r,:));
    eb.Color = zeros(1,3);
    eb.LineStyle = 'none';
    set(get(get(eb, 'Annotation'), 'LegendInformation'), 'IconDisplayStyle', 'off');
end

%for plotting no aTc, no IPTG condition (b8)
% for r = 1:8
```

```
%     eb = errorbar(t-0.35 + (r-1)*0.1, bg(:,r), error(r,:));
%     eb.Color = zeros(1,3);
%     eb.LineStyle = 'none';
%     set(get(get(eb, 'Annotation'), 'LegendInformation'), 'IconDisplayStyle', 'off');
% end

%% Figure 4c
% QUAS-T7-TetO-GFP + T7-LacO-QF-T7-TetO-TetR 10 hours
clear

data = [260 277 264 250 246 263 256 255 240 226
264 293 273 257 249 250 244 231 217 202
269 288 274 250 247 246 237 241 232 216
540 484 376 343 329 328 322 315 286 279
592 552 426 359 334 318 312 288 271 243
502 477 385 348 314 312 306 288 271 262
1351    1061    566 445 409 395 381 360 342 302
1540    1306    671 471 421 390 369 363 310 278
1461    1209    617 449 410 384 378 356 331 311
8343    4714    1314    830 710 652 604 571 509 470
8414    7509    2274    1095    823 708 628 567 486 435
8426    7783    2818    1239    873 746 665 611 562 500
11086   18887   6741    2361    1761    1550    1342    1204    1083    973
9810    21385   10706   3148    1983    1704    1440    1253    1071    917
9576    21672   12975   3971    2462    1988    1720    1515    1260    984
10106   19349   9043    3538    2400    2021    1766    1596    1396    1209
8909    20890   14310   5480    3071    2440    2152    1739    1478    1214
8849    21025   15676   7245    4007    3096    2662    2255    1968    1560
4480    10268   8746    5238    4220    4117    3769    3416    3091    2577
4126    15137   15424   8675    5949    5291    4311    3540    2961    2434
4428    13848   12182   7515    5681    4931    4389    3751    3426    2991];

% means of triplicates
data_means = [264.3333 286 270.3333 252.3333 247.3333 253 245.6667 242.3333 229.6667
214.6667
544.6667 504.3333 395.6667 350 325.6667 319.3333 313.3333 297 276 261.3333
1450.667 1192 618 455 413.3333 389.6667 376 359.6667 327.6667 297
8394.333 6668.667 2135.333 1054.667 802 702 632.3333 583 519 468.3333
10157.33 20648 10140.67 3160 2068.667 1747.333 1500.667 1324 1138 958
9288 20421.33 13009.67 5421 3159.333 2519 2193.333 1863.333 1614 1327.667
4344.667 13084.33 12117.33 7142.667 5283.333 4779.667 4156.333 3569 3159.333 2667.333
178 174 344.3333 244.3333 186 130 145 154.3333 127.2667 136 ];

%standard deviation
error = [3.681787006    6.683312552 4.496912521 3.299831646 1.247219129 7.257180352
7.845734864 9.843215373 9.533566431 9.843215373
36.89022755 33.82635396 21.76133166 6.683312552 8.498365856 6.599663291 6.599663291
12.72792206 7.071067812 14.70449667
77.50412175 100.740591  42.87190222 11.43095213 5.436502143 4.496912521 5.099019514
2.867441756 13.27487183 13.92838828
36.62725154 1386.677163 621.7852434 169.3917222 68.18113131 38.60915263 25.09094569
19.86621923 31.82242396 26.56229575
663.578849  1250.715262 2576.223377 657.3345166 292.5227437 181.4190974 160.1693548
136.5308268 86.40601831 29.33712097
```

```
578.9317749 760.2544896 2859.757837 1513.951342 659.0216655 442.4078058 366.9562493
283.0363149 252.5443855 164.2971563
156.0712088 2059.802633 2726.665526 1427.635886 759.8088941 491.0840616 275.7309478
138.2919617 195.8882902 236.1953053
9.797958971 5.099019514 5.792715732 6.944222219 11.22497216 5.715476066 11.86029792
1.247219129 21.60576055 3.559026084];

b1 = data_means(1,:);
b2 = data_means(2,:);
b3 = data_means(3,:);
b4 = data_means(4,:);
b5 = data_means(5,:);
b6 = data_means(6,:);
b7 = data_means(7,:);
b8 = data_means(8,:);

bg = [b1(:), b2(:), b3(:), b4(:), b5(:), b6(:), b7(:)];
t = 1:10;
time_points = [];
for hour = 1:length(t)
    time_temp = [];
    for sample = 1:7
        temp = ones(1,3);
        multiplier = hour - 0.345 + (sample-1)*0.115;
        temp = temp.* multiplier;
        time_temp = [time_temp, temp];
    end
    time_points = [time_points, time_temp];
end

data_points = data(:)';

figure
hold on
bar(t, bg, 'grouped')
plot(time_points, data_points, '.k')
title('QUAS-T7-TetO-GFP + T7-LacO-QF-T7-TetO-TetR (10Hr)')
xlabel('Time (Hr)')
ylabel('Mean Fluorescent Intensity (AU)')
h = legend('0', '30', '50', '100', '300', '500', '700');
title(h, 'aTc (ng/mL)')
% set(gca, 'YScale', 'log');
axis([0, 11, 0, 25000]);

for r = 1:7
    eb = errorbar(t-0.345 + (r-1)*0.115, bg(:,r), error(r,:));
    eb.Color = zeros(1,3);
    eb.LineStyle = 'none';
    set(get(get(eb, 'Annotation'), 'LegendInformation'), 'IconDisplayStyle', 'off');
end

%code for plotting errorbars on no IPTG, no aTc condition (b8)
% for r = 1:8
%     eb = errorbar(t-.35 + (r-1)*0.1, bg(:,r), error(r,:));
```

```
%     eb.Color = zeros(1,3);
%     eb.LineStyle = 'none';
%     set(get(get(eb, 'Annotation'), 'LegendInformation'), 'IconDisplayStyle', 'off');
% end

%% Figure 4d
% QUAS-T7-TetO-GFP_T7-LacO-TetR & T7-LacO-QF "Colony B"
clear

data_full = [260    277 264 250 246 263 256 255 240 226
264 293 273 257 249 250 244 231 217 202
269 288 274 250 247 246 237 241 232 216
540 484 376 343 329 328 322 315 286 279
592 552 426 359 334 318 312 288 271 243
502 477 385 348 314 312 306 288 271 262
1351    1061    566 445 409 395 381 360 342 302
1540    1306    671 471 421 390 369 363 310 278
1461    1209    617 449 410 384 378 356 331 311
8343    4714    1314    830 710 652 604 571 509 470
8414    7509    2274    1095    823 708 628 567 486 435
8426    7783    2818    1239    873 746 665 611 562 500
11086   18887   6741    2361    1761    1550    1342    1204    1083    973
9810    21385   10706   3148    1983    1704    1440    1253    1071    917
9576    21672   12975   3971    2462    1988    1720    1515    1260    984
10106   19349   9043    3538    2400    2021    1766    1596    1396    1209
8909    20890   14310   5480    3071    2440    2152    1739    1478    1214
8849    21025   15676   7245    4007    3096    2662    2255    1968    1560
4480    10268   8746    5238    4220    4117    3769    3416    3091    2577
4126    15137   15424   8675    5949    5291    4311    3540    2961    2434
4428    13848   12182   7515    5681    4931    4389    3751    3426    2991];

%data used for figure
data = [14399   47866   65349   72396   61972   43597   31390   16417   9231    4842
25300   72474   93205   93230   66107   42004   24953   13589   7096    3624
27499   79283   100187  94008   61781   36366   21600   13433   7244    3777
17622   58518   81267   86969   74937   54045   36962   20235   10805   5780
21231   67363   89234   89748   70719   45383   26343   15539   8291    4480
26481   74621   97222   88945   63081   39283   25027   14016   7515    4054
14999   52891   75450   86496   78207   62852   42096   25500   14143   7231
20000   61141   81917   92117   74961   53643   32067   18734   10016   5341
22743   68555   89797   79988   76310   50559   32100   19162   10741   5996
5724    37182   56792   66837   72434   63785   49979   32731   19714   11393
11157   45091   65302   75143   74164   58817   42454   26592   15737   10124
13625   52606   71318   81853   77488   61824   42572   28292   15957   9869];

% means of triplicates
data_means = [22399.33333   66541   86247   86544.66667 63286.66667 40655.66667 25981
14479.66667 7857    4081
22155.66667 70740.33333 85303   92270.33333 68068.33333 45481.66667 28349.33333 15827
8268.666667 4435
22412   69567.33333 92339.33333 91710.33333 68319.66667 45094.33333 28326.33333
15794.33333 8410.333333 4176.666667
21778   66834   89241   88554   69579   46237   29444   16596.66667 8870.333333 4771.333333
```

```
19247.33333 60862.33333 82388   86200.33333 76492.66667 55684.66667 35421   21132
11633.33333 6189.333333
10168.66667 44959.66667 64470.66667 74611   74695.33333 61475.33333 45001.66667 29205
17136   10462
3455.666667 26119.33333 40660   47636.33333 47660.33333 44510.33333 34844.33333
24460.66667 14999.66667 10155.66667];

error = [7015.190684    16527.47225 18431.81716 12259.27801 2444.346607 3799.3871
4975.300292 1679.592014 1192.217681 663.4704213
3990.437361 11415.50736 15225.93537 4106.118402 3493.925634 3486.479791 5182.818281
2700.908921 1329.899746 669.4602303
3928.290595 8541.399554 8678.75304  4512.721389 6187.533946 5293.893306 4688.995024
3256.513678 1490.400729 585.7860815
4454.758916 8064.523111 7977.502303 1430.164676 6009.649241 7417.961175 6543.9443
3241.602124 1719.809679 899.1247596
3926.482438 7835.717283 7185.087543 6069.903157 1630.691367 6395.761435 5780.743118
3788.847318 2203.457813 959.7178405
4042.159365 7712.838669 7298.596121 7522.122839 2568.55316  2502.285422 4310.900873
3169.701405 2235.321677 816.2885519
472.0872095 4374.306612 6216.209778 7727.192526 5339.315156 4646.04416  2346.465498
2272.676909 1203.154326 802.0737705];

b1 = data_means(1,:);
b2 = data_means(2,:); %these data not presented in figure
b3 = data_means(3,:); %these data not presented in figure
b4 = data_means(4,:);
b5 = data_means(5,:);
b6 = data_means(6,:);
b7 = data_means(7,:); %these data not presented in figure

bg = [b1(:), b4(:), b5(:), b6(:)];
t = 1:10;
time_points = [];
for hour = 1:length(t)
    time_temp = [];
    for sample = 1:4
        temp = ones(1,3);
        multiplier = hour - 0.28 + (sample-1)*0.185;
        temp = temp.* multiplier;
        time_temp = [time_temp, temp];
    end
    time_points = [time_points, time_temp];
end

data_points = data(:)';

figure
hold on
bar(t, bg, 'grouped')
plot(time_points, data_points, '.k')
title({'Biosensor & T7-LacO-QF', 'Figure 4d iii'})
xlabel('Time (Hr)')
ylabel('Mean Fluorescent Intensity (AU)')
h = legend('0', '100', '300', '500');
```

```
title(h, 'aTc (ng/mL)')

for r = 1:4
    eb = errorbar(t-0.28 + (r-1)*0.185, bg(:,r), error(r,:));
    eb.Color = zeros(1,3);
    eb.LineStyle = 'none';
    set(get(get(eb, 'Annotation'), 'LegendInformation'), 'IconDisplayStyle', 'off');
end
```

Supplementary Software Figure 4: Matlab code for generating all of the plots in Figure 4: Genetic devices built with components from the Q system couple with the TetR system.

```
% Code for Supplementary Figure 2.

%Fluorescence data
%each row represents a new sample, each column is a new time point
clear
data = [3722.279103 450.1227701 304.4238758 313.896902  341.1591525
4969.576401 720.1339886 335.1124722 324.8617682 341.562218
5056.501663 730.0188282 320.9268416 313.4007202 352.060779
61.39772727 60.79465358 61.57972323 56.58043084 63.11540008
48.62328558 54.06290629 53.29075868 55.03034235 61.12081412
54.2048314  52.99799639 57.79292624 60.19625316 64.66404358
3624.089255 5126.168765 613.0413545 191.3913087 184.3694198
3662.938063 5251.855888 2499.573598 246.0711076 205.9403074
3616.874713 5457.328266 1695.459894 211.1602756 190.9170323
75.62803289 63.23449729 58.21890598 50.42962439 43.3650994
68.50155513 65.97180846 56.53258019 52.86761816 37.47852041
67.97495638 68.36446355 48.85301057 66.96711858 49.19531572];
norm = mean(data(1:3, 1));
data = data./norm;

T7 = data(1:3, :);
QT = data(4:6, :);
QTqf = data(7:9, :);
BL21 = data(10:12, :);

b1 = mean(T7);
b2 = mean(QT);
b3 = mean(QTqf);
b4 = mean(BL21);

e1 = std(T7);
e2 = std(QT);
e3 = std(QTqf);
e4 = std(BL21);

error = [e1(:), e2(:), e3(:), e4(:)];

bg = [b1(:), b2(:), b3(:), b4(:)];
t = [2, 4, 6,8, 10];

time_points = [];
for hour = 1:length(t)
    time_temp = [];
    for sample = 1:4
        temp = ones(1,3);
        multiplier = t(hour) - 0.55 + (sample-1)*0.365;
        temp = temp.* multiplier;
        time_temp = [time_temp, temp];
    end
    time_points = [time_points, time_temp];
end

data_points = data(:)';
```

```
figure
hold on
bar(t, bg, 'grouped')
plot(time_points, data_points, '.k')
set(gca, 'Yscale', 'log')
ylim([1e-3, 10])
ylabel('Normalized GFP Fluorescence')
xlabel('Time (hours)')
title('Supplemental 2')
legend('T7', 'QUAS-0-T7', 'QUAS-0-T7 + QF', 'BL21')
xticks(t)
yticks([1e-3, 1e-2, 0.1, 1, 10])
yticklabels({'0.001', '0.01', '0.1', '1', '10'})

for r = 1:4
    eb = errorbar(t-0.55 + (r-1)*0.365, bg(:,r), error(:, r));
    eb.Color = zeros(1,3);
    eb.LineStyle = 'none';
    set(get(get(eb, 'Annotation'), 'LegendInformation'), 'IconDisplayStyle', 'off');
end
```

Supplementary Software Figure 5: Matlab code for generating all of the plots in Supplementary Figure 2: : T7 expression and untransformed BL21 (DE3) cell lines.

```
%Code for Supplemental Figure 3

%each column is a new timepoint
clear
data = [0.946428571 7.678571429 9.107142857 9.464285714 10.71428571
0.857142857 7.321428571 11.78571429 10.53571429 10
1.196428571 5.892857143 9.464285714 11.25   10.53571429
1.03030303  3.787878788 0.363636364 0.009393939 0.001515152
0.984848485 2.575757576 0.181818182 0.010151515 0.03030303
0.984848485 4.090909091 0.303030303 0.010606061 0.033333333];
data = data.*100; %convert to %CFU/mL

ccdb = data(1:3, :);
ccdbQF = data(4:6, :);

%mean
b1 = mean(ccdb);
b2 = mean(ccdbQF);

%standard deviation
e1 = std(ccdb);
e2 = std(ccdbQF);

t = 0:4; %hrs

time_points1 = [];

for r = 1:length(t)
    temp = ones(1,3).*t(r);
    time_points1 = [time_points1, temp];
end

time_points2 = [];
for r = 1:length(t)
    temp = ones(1,3).*t(r);
    time_points2 = [time_points2, temp];
end

%data for conditions without IPTG
data_noIPTG = [6.964285714  10.53571429 11.96428571 10.89285714
7.321428571 8.75    11.96428571 13.21428571
8.928571429 8.214285714 11.90476191 12.20238095
7.575757576 9.545454545 24.24242424 18.18181818
9.090909091 13.03030303 22.72727273 15.15151515
8.03030303  12.12121212 24.24242424 12.12121212];
data_noIPTG = data_noIPTG.*100; %convert decimal to %CFU/mL

ccdb_no = data_noIPTG(1:3,:);
ccdbQF_no = data_noIPTG(4:6,:);

b1_no = mean(ccdb_no);
b2_no = mean(ccdbQF_no);

e1_no = std(ccdb_no);
```

```
e2_no = std(ccdbQF_no);

t_no = 1:4;
time_points_no1 = [];

for r=1:length(t_no)
    temp = ones(1,3).*t_no(r);
    time_points_no1 = [time_points_no1, temp];
end

time_points_no2 = [];
for r=1:length(t_no)
    temp = ones(1,3).*t_no(r);
    time_points_no2 = [time_points_no2, temp];
end

figure
hold on
plot(t, b1, 'k')
plot(t, b2, 'c')
p3 = plot(t_no, b1_no);
p3.Color = [0.8500, 0.3250, 0.0980];
p4 = plot(t_no, b2_no);
p4.Color = [0.9290, 0.6940, 0.1250];

%Plot data points
plot(time_points1, ccdb(:), '.k')
plot(time_points2, ccdbQF(:), '.c')
p3 = plot(time_points_no1, ccdb_no(:), '.');
p3.Color = [0.8500, 0.3250, 0.0980];
p4 = plot(time_points_no2, ccdbQF_no(:), '.');
p4.Color = [0.9290, 0.6940, 0.1250];

ylim([0.1 1e4])
set(gca, 'YScale', 'log')
yticks([0.1, 1, 10, 100, 1e3, 1e4])
yticklabels({'0.1', '1', '10', '100', '1000', '10000'})
xticks([0:4])
xlabel('Time (hours)')
ylabel('%CFU/mL')
h = legend('CcdB', 'CcdB + QF', 'CcdB -IPTG', 'CcdB + QF -IPTG');
set(h, 'Location', 'East')
title('Sumpplemental Figure 3')

%errorbars
eb1 = errorbar(t, b1, e1);
eb1.Color = zeros(1,3);
eb.LineStyle = 'none';
set(get(get(eb1, 'Annotation'), 'LegendInformation'), 'IconDisplayStyle', 'off');

eb2 = errorbar(t, b2, e2);
eb2.Color = 'c';
eb2.LineStyle = 'none';
set(get(get(eb2, 'Annotation'), 'LegendInformation'), 'IconDisplayStyle', 'off');
```

```
eb1_no = errorbar(t_no, b1_no, e1_no);
eb1_no.Color = [0.8500, 0.3250, 0.0980];
eb1_no.LineStyle = 'none';
set(get(get(eb1_no, 'Annotation'), 'LegendInformation'), 'IconDisplayStyle', 'off');

eb2_no = errorbar(t_no, b2_no, e2_no);
eb2_no.Color = [0.9290, 0.6940, 0.1250];
eb2_no.LineStyle = 'none';
set(get(get(eb2_no, 'Annotation'), 'LegendInformation'), 'IconDisplayStyle', 'off');
```

Supplementary Software Figure 6: Matlab code for generating all of the plots in Supplementary Figure 3: CcdB induced death including uninduced controls.

```
%Code for supplemental Figure 5% T7-TetO-GFP & T7-LacO-TetR

clear

%Mean fluorescent intensity
data = [290 253 251 245 215 171 148 129 134 190
270 243 235 238 227 193 184 165 153 140
269 237 231 233 216 202 189 180 188 171
534 420 359 321 253 193 157 128 149 230
472 371 321 306 274 234 213 185 180 162
429 336 296 273 256 246 225 207 198 203
656 499 418 359 294 203 158 138 152 215
591 459 375 347 313 252 224 198 200 173
514 398 343 300 277 248 226 213 208 217
954 657 509 411 313 238 165 128 168 215
867 596 471 393 344 281 257 216 201 172
711 497 390 342 299 266 247 218 232 214
2368    1533    1102    872 661 417 311 206 187 230
2205    1433    974 831 700 603 558 413 344 275
2093    1320    978 775 646 586 493 473 412 509
2991    2984    2064    1497    1150    711 473 289 225 245
2837    2815    1905    1440    1187    1033    874 744 561 438
2877    2699    1881    1399    1194    1066    928 877 710 600
3027    3918    2457    1825    1361    889 539 347 203 225
2931    3646    2519    1781    1480    1265    1130    931 688 499
2915    3528    2415    1736    1456    1282    1177    994 877 733];

% means of triplicates
data_means = [276.3333333   244.3333333 239 238.6666667 219.3333333 188.6666667
173.6666667 158 158.3333333 167
478.3333333 375.6666667 325.3333333 300 261 224.3333333 198.3333333 173.3333333
175.6666667 198.3333333
587 452 378.6666667 335.3333333 294.6666667 234.3333333 202.6666667 183 186.6666667
201.6666667
844 583.3333333 456.6666667 382 318.6666667 261.6666667 223 187.3333333 200.3333333
200.3333333
2222    1428.666667 1018    826 669 535.3333333 454 364 314.3333333 338
2901.666667 2832.666667 1950    1445.333333 1177    936.6666667 758.3333333 636.6666667
498.6666667 427.6666667
2957.666667 3697.333333 2463.666667 1780.666667 1432.333333 1145.333333 948.6666667
757.3333333 589.3333333 485.6666667];

error = [11.8462371 8.082903769 10.58300524 6.027713773 6.658328118 15.94783162 22.36813209
26.21068484 27.39221301 25.23885893
52.78573039 42.19399641 31.72275734 24.55605832 11.35781669 27.7908858  36.2950869
40.77172223 24.78574859 34.23935358
71.08445681 50.8625599  37.6342043  31.18225991 18.00925688 27.20906712 38.69539163
39.68626967 30.28751118 24.84619354
123.1218908 80.748581   60.78102774 35.79106034 23.02896727 21.82506205 50.47771786
51.39390366 32.00520791 24.54248018
138.2859357 106.5660984 72.77362159 48.6929153  27.87471973 102.8315775 128.0351514
140.0821188 115.3964182 149.7898528
79.90828076 143.3189915 99.45350673 49.21720566 23.64318084 196.1283593 248.5766146
308.3445043 248.4357731 177.7254437
```

```
60.57502236 200.0033333 52.31953109 44.50093632 62.93117934 222.1538506 355.559184
356.7524819 347.6641099 254.2623317];

b1 = data_means(1,:);
b2 = data_means(2,:);
b3 = data_means(3,:);
b4 = data_means(4,:);
b5 = data_means(5,:);
b6 = data_means(6,:);
b7 = data_means(7,:);

bg = [b1(:), b2(:), b3(:), b4(:), b5(:), b6(:), b7(:)];
t = 1:10;

time_points = [];
for hour = 1:length(t)
    time_temp = [];
    for sample = 1:7
        temp = ones(1,3);
        multiplier = hour - 0.345 + (sample-1)*0.115;
        temp = temp.* multiplier;
        time_temp = [time_temp, temp];
    end
    time_points = [time_points, time_temp];
end

data_points = data(:)';

figure
hold on
bar(t, bg, 'grouped')
plot(time_points, data_points, '.k')
title('T7-TetO-GFP & T7-LacO-TetR')
xlabel('Time (Hr)')
ylabel('Mean Fluorescent Intensity (AU)')
h = legend('0', '30', '50', '100', '300', '500', '700');
title(h, 'aTc (ng/mL)')
% axis([0, 11, 0, 2.5e4]);

for r = 1:7
    eb = errorbar(t-0.345 + (r-1)*0.115, bg(:,r), error(r,:));
    eb.Color = zeros(1,3);
    eb.LineStyle = 'none';
    set(get(get(eb, 'Annotation'), 'LegendInformation'), 'IconDisplayStyle', 'off');
end

%% Statistical Analysis
% Dosage Data from 8-31-20
% T7-TetO-GFP & T7-LacO-TetR

%data is organized as follows:
%Columns indicate time (hr) 1-10
%rows indicate aTc condition (ng/mL)
%rows 1-3: aTc 0
```

```
%4-6: aTc 30
%7-9: aTc 50
%10-12: aTc 100
%13-15: aTc 300
%16-18: aTc 500
%19-21: aTc 700

data = [290 253 251 245 215 171 148 129 134 190
270 243 235 238 227 193 184 165 153 140
269 237 231 233 216 202 189 180 188 171
534 420 359 321 253 193 157 128 149 230
472 371 321 306 274 234 213 185 180 162
429 336 296 273 256 246 225 207 198 203
656 499 418 359 294 203 158 138 152 215
591 459 375 347 313 252 224 198 200 173
514 398 343 300 277 248 226 213 208 217
954 657 509 411 313 238 165 128 168 215
867 596 471 393 344 281 257 216 201 172
711 497 390 342 299 266 247 218 232 214
2368    1533    1102    872 661 417 311 206 187 230
2205    1433    974 831 700 603 558 413 344 275
2093    1320    978 775 646 586 493 473 412 509
2991    2984    2064    1497    1150    711 473 289 225 245
2837    2815    1905    1440    1187    1033    874 744 561 438
2877    2699    1881    1399    1194    1066    928 877 710 600
3027    3918    2457    1825    1361    889 539 347 203 225
2931    3646    2519    1781    1480    1265    1130    931 688 499
2915    3528    2415    1736    1456    1282    1177    994 877 733];

for hour = 1:10
    count = 1;
    for condition = 1:3:18
        interval = condition +3;
        while interval <21
            h = 3; % if 3 appears in results or pvalues, there was an error with ttest2
            p = 3;
            [h, p] = ttest2(data(condition:condition+2,hour), data(interval:interval+2, hour));
            results(count, hour) = h;
            pvalues(count, hour) = p;
            interval = interval + 3;
            count = count + 1;
        end
    end
end
```
Supplementary Software Figure 7: Matlab code for generating all of the plots in Supplementary Figure 5: Traditional TetR system.

```
% Code for Supplemental Figure 6: Growth curves of BL21 E. coli

%% Supplementary Figure 6a
clear

minus5 = [0.092 0.089   0.09
0.095   0.097   0.099
0.113   0.118   0.114
0.141   0.151   0.167
0.238   0.229   0.243
0.353   0.372   0.346
0.428   0.442   0.511
0.548   0.518   0.532
0.497   0.579   0.61
0.618   0.634   0.638
0.673   0.652   0.68
0.647   0.681   0.716
0.878   0.729   0.671
0.686   0.793   0.675
0.718   0.755   0.713
0.786   0.816   0.757
0.783   0.842   0.77
0.886   0.907   0.843
0.736   0.802   0.815
0.841   0.966   0.959
0.875   0.857   0.999];

minus10 = [0.081    0.087   0.082
0.081   0.085   0.084
0.094   0.099   0.094
0.118   0.122   0.115
0.204   0.221   0.177
0.275   0.287   0.272
0.452   0.43    0.409
0.492   0.489   0.511
0.577   0.58    0.581
0.582   0.664   0.648
0.592   0.607   0.669
0.71    0.793   0.698
0.721   0.801   0.764
0.814   0.863   0.847
0.849   0.862   0.81
0.874   0.993   0.91
0.975   0.943   0.922
1.084   0.901   0.999
1.165   1.069   0.998
1.056   1.005   1.035
1.099   1.096   1.22];

minus15 = [0.089    0.088   0.087
0.095   0.095   0.094
0.112   0.127   0.124
0.152   0.157   0.161
0.242   0.262   0.248
```

```
0.345   0.374   0.386
0.406   0.43    0.467
0.526   0.539   0.519
0.514   0.554   0.589
0.598   0.642   0.575
0.625   0.639   0.648
0.567   0.585   0.748
0.647   0.757   0.663
0.627   0.627   0.75
0.714   0.733   0.767
0.717   0.802   0.777
0.763   0.799   0.803
0.73    0.83    0.798
0.818   0.808   0.98
0.815   0.85    0.967
0.911   0.981   0.942];

BL21 = [0.099   0.104   0.096
0.103   0.104   0.096
0.137   0.136   0.121
0.189   0.213   0.179
0.267   0.277   0.256
0.397   0.465   0.362
0.457   0.47    0.468
0.574   0.564   0.537
0.617   0.642   0.67
0.66    0.641   0.62
0.698   0.712   0.732
0.736   0.756   0.764
0.801   0.804   0.841
0.883   0.871   0.836
0.876   0.887   0.882
0.918   0.924   0.915
0.982   0.978   0.894
0.994   1.004   0.993
0.973   1   0.996
0.955   1.062   1.031
1.119   1.14    1.049];

data_upstream = {minus15, minus10, minus5, BL21};

for condition = 1:length(data_upstream)
    tempdata = data_upstream{condition};
    tempdata = tempdata - 0.046; %account for diffraction by pure LB and antibiotics
    averages_upstream{condition} = mean(tempdata'); %average OD for each timepoint
    error_upstream{condition} = std(tempdata'); %standard deviation for each timepoint
end

t = 0:0.5:0.5*(length(minus5)-1);
figure
hold on
for r = 1:length(averages_upstream)
    plot(t, averages_upstream{r}, '-o')
    eb1 = errorbar(t, averages_upstream{r}, error_upstream{r});
```

```
    eb1.Color = [0 0 0];
    eb1.LineStyle = 'none';
    set(get(get(eb1, 'Annotation'), 'LegendInformation'), 'IconDisplayStyle', 'off');
end
xlabel('Time (Hrs)')
ylabel('OD_{600}')
h = legend('QUAS-15-T7-GFP', 'QUAS-10-T7-GFP', 'QUAS-5-T7-GFP', 'No Plasmid');
set(h, 'Location', 'NorthWest');
title('Upstream Constructs')

%% Supplemental Figure 6b
clear

plus5 = [0.081  0.081   0.082
0.08    0.085   0.084
0.097   0.098   0.099
0.119   0.118   0.111
0.174   0.183   0.164
0.254   0.259   0.28
0.414   0.4 0.368
0.476   0.511   0.584
0.582   0.6 0.574
0.599   0.685   0.636
0.612   0.652   0.648
0.711   0.738   0.66
0.697   0.665   0.781
0.771   0.781   0.76
0.764   0.773   0.736
0.768   0.778   0.791
0.821   0.889   0.808
0.774   0.792   0.854
0.832   1.013   0.946
0.961   0.984   1.014
0.998   1   1.058];

plus10 = [0.08  0.081   0.079
0.084   0.086   0.084
0.093   0.097   0.095
0.115   0.115   0.116
0.182   0.167   0.168
0.28    0.298   0.319
0.4 0.413   0.402
0.516   0.529   0.526
0.57    0.582   0.585
0.621   0.676   0.583
0.627   0.599   0.625
0.636   0.65    0.654
0.688   0.675   0.702
0.757   0.734   0.756
0.746   0.763   0.731
0.822   0.793   0.77
0.87    0.919   0.931
0.826   0.925   0.94
0.93    0.88    1.043
```

```
0.947   1.024   0.975
0.909   0.962   1.02];

plus15 = [0.089 0.089   0.09
0.097   0.095   0.094
0.109   0.108   0.107
0.161   0.153   0.149
0.242   0.217   0.227
0.345   0.338   0.319
0.465   0.392   0.41
0.511   0.479   0.45
0.612   0.591   0.552
0.648   0.555   0.534
0.721   0.592   0.543
0.76    0.623   0.642
0.804   0.664   0.612
0.874   0.757   0.66
0.894   0.797   0.779
0.964   0.743   0.765
0.932   0.837   0.851
0.961   0.801   0.855
0.968   1.028   0.835
1.028   1.09    1.002
1.136   0.942   1.048];

BL21 = [0.099   0.104   0.096
0.103   0.104   0.096
0.137   0.136   0.121
0.189   0.213   0.179
0.267   0.277   0.256
0.397   0.465   0.362
0.457   0.47    0.468
0.574   0.564   0.537
0.617   0.642   0.67
0.66    0.641   0.62
0.698   0.712   0.732
0.736   0.756   0.764
0.801   0.804   0.841
0.883   0.871   0.836
0.876   0.887   0.882
0.918   0.924   0.915
0.982   0.978   0.894
0.994   1.004   0.993
0.973   1   0.996
0.955   1.062   1.031
1.119   1.14    1.049];

data_downstream = {plus15, plus10, plus5, BL21};

for condition = 1:length(data_downstream)
    tempdata = data_downstream{condition};
    tempdata = tempdata - 0.046; %account for diffraction by pure LB and antibiotics
    averages_downstream{condition} = mean(tempdata'); %average OD for each timepoint
    error_downstream{condition} = std(tempdata'); %standard deviation for each timepoint
```

```
end

t = 0:0.5:0.5*(length(plus5)-1);
figure
hold on
for r = 1:length(averages_downstream)
    plot(t, averages_downstream{r}, '-o')
    eb1 = errorbar(t, averages_downstream{r}, error_downstream{r});
    eb1.Color = [0 0 0];
    eb1.LineStyle = 'none';
    set(get(get(eb1, 'Annotation'), 'LegendInformation'), 'IconDisplayStyle', 'off');
end
xlabel('Time (Hrs)')
ylabel('OD_{600}')
h = legend('T7-15-QUAS-GFP', 'T7-10-QUAS-GFP', 'T7-5-QUAS-GFP', 'No Plasmid');
set(h, 'Location', 'NorthWest');
title('Downstream Constructs')

%% Supplemental Figure 6c
clear

minus5QF = [0.075   0.076   0.079
0.074   0.085   0.077
0.080   0.094   0.084
0.106   0.130   0.103
0.145   0.201   0.117
0.209   0.273   0.139
0.325   0.364   0.246
0.409   0.505   0.371
0.498   0.59    0.494
0.529   0.646   0.549
0.583   0.724   0.613
0.677   0.708   0.672
0.726   0.755   0.685
0.75    0.813   0.733
0.69    0.821   0.796
0.797   0.823   0.797
0.798   0.864   0.816
0.846   0.827   0.864
0.79    0.839   0.814
0.822   0.847   0.828
0.833   0.872   0.813];

minus10QF = [0.073  0.07    0.07
0.076   0.075   0.075
0.075   0.073   0.073
0.079   0.078   0.077
0.086   0.087   0.088
0.113   0.12    0.108
0.148   0.157   0.162
0.251   0.241   0.228
0.298   0.336   0.343
0.393   0.404   0.392
0.487   0.503   0.464
```

```
0.516   0.502   0.516
0.557   0.547   0.56
0.603   0.663   0.589
0.603   0.62    0.635
0.684   0.741   0.66
0.706   0.729   0.733
0.683   0.674   0.642
0.714   0.632   0.614
0.707   0.669   0.672
0.772   0.777   0.719];

minus15QF = [0.072  0.073   0.092
    0.085   0.075   0.075
0.075   0.081   0.081
0.091   0.097   0.094
0.112   0.13    0.117
0.155   0.185   0.171
0.243   0.268   0.261
0.338   0.384   0.356
0.447   0.509   0.435
0.527   0.567   0.527
0.613   0.627   0.553
0.595   0.689   0.592
0.63    0.706   0.676
0.7 0.725   0.653
0.747   0.82    0.723
0.766   0.867   0.755
0.755   0.82    0.797
0.835   0.816   0.773
0.794   0.816   0.785
0.866   0.858   0.872
0.819   0.877   0.825];

BL21 = [0.099   0.104   0.096
0.103   0.104   0.096
0.137   0.136   0.121
0.189   0.213   0.179
0.267   0.277   0.256
0.397   0.465   0.362
0.457   0.47    0.468
0.574   0.564   0.537
0.617   0.642   0.67
0.66    0.641   0.62
0.698   0.712   0.732
0.736   0.756   0.764
0.801   0.804   0.841
0.883   0.871   0.836
0.876   0.887   0.882
0.918   0.924   0.915
0.982   0.978   0.894
0.994   1.004   0.993
0.973   1   0.996
0.955   1.062   1.031
1.119   1.14    1.049];
```

```
data_upstreamQF = {minus15QF, minus10QF, minus5QF, BL21};

for condition = 1:length(data_upstreamQF)
    tempdata = data_upstreamQF{condition};
    tempdata = tempdata - 0.046; %account for diffraction by pure LB and antibiotics
    averages_upstreamQF{condition} = mean(tempdata'); %average OD for each timepoint
    error_upstreamQF{condition} = std(tempdata'); %standard deviation for each timepoint
end

% t = 0:0.5:0.5*(length(minus5QF)-1);
t = 0:0.5:10;
figure
hold on
for r = 1:length(averages_upstreamQF)
    plot(t, averages_upstreamQF{r}, '-o')
    eb1 = errorbar(t, averages_upstreamQF{r}, error_upstreamQF{r});
    eb1.Color = [0 0 0];
    eb1.LineStyle = 'none';
    set(get(get(eb1, 'Annotation'), 'LegendInformation'), 'IconDisplayStyle', 'off');
    end
xlabel('Time (Hrs)')
ylabel('OD_{600}')
h = legend('QUAS-15-T7-GFP + QF', 'QUAS-10-T7-GFP + QF', 'QUAS-5-T7-GFP + QF', 'No Plasmid');
set(h, 'Location', 'NorthWest');
title('Upstream Constructs with QF')

%% Supplemental Figure 6d
clear

plus5QF = [0.075    0.071   0.071
0.075   0.076   0.076
0.082   0.075   0.074
0.085   0.087   0.079
0.093   0.092   0.086
0.125   0.12    0.1
0.173   0.195   0.157
0.28    0.259   0.209
0.379   0.367   0.317
0.393   0.419   0.372
0.488   0.469   0.436
0.532   0.49    0.52
0.566   0.569   0.551
0.589   0.555   0.655
0.623   0.601   0.632
0.671   0.607   0.666
0.632   0.741   0.653
0.735   0.72    0.703
0.703   0.658   0.696
0.708   0.72    0.662
0.719   0.674   0.693];

plus10QF = [0.071   0.07    0.074
0.073   0.073   0.071
```

```
0.079   0.08    0.075
0.09    0.088   0.083
0.114   0.103   0.095
0.146   0.138   0.127
0.219   0.212   0.182
0.315   0.314   0.206
0.393   0.377   0.353
0.517   0.476   0.446
0.56    0.521   0.55
0.589   0.575   0.591
0.628   0.601   0.611
0.664   0.636   0.646
0.709   0.716   0.692
0.669   0.701   0.752
0.797   0.736   0.779
0.752   0.753   0.825
0.781   0.77    0.792
0.791   0.808   0.817
0.817   0.82    0.861];

plus15QF = [0.072   0.073   0.07
0.072   0.074   0.073
0.076   0.073   0.075
0.077   0.077   0.079
0.083   0.083   0.083
0.098   0.101   0.099
0.109   0.12    0.135
0.174   0.167   0.154
0.213   0.276   0.283
0.34    0.352   0.351
0.396   0.441   0.551
0.553   0.536   0.509
0.566   0.562   0.523
0.541   0.555   0.622
0.65    0.596   0.588
0.647   0.632   0.724
0.601   0.606   0.63
0.632   0.635   0.625
0.738   0.61    0.702
0.705   0.721   0.735
0.721   0.71    0.731];

BL21 = [0.099   0.104   0.096
0.103   0.104   0.096
0.137   0.136   0.121
0.189   0.213   0.179
0.267   0.277   0.256
0.397   0.465   0.362
0.457   0.47    0.468
0.574   0.564   0.537
0.617   0.642   0.67
0.66    0.641   0.62
0.698   0.712   0.732
0.736   0.756   0.764
```

```
0.801	0.804	0.841
0.883	0.871	0.836
0.876	0.887	0.882
0.918	0.924	0.915
0.982	0.978	0.894
0.994	1.004	0.993
0.973	1	0.996
0.955	1.062	1.031
1.119	1.14	1.049];

data_downstreamQF = {plus15QF, plus10QF, plus5QF, BL21};

for condition = 1:length(data_downstreamQF)
    tempdata = data_downstreamQF{condition};
    tempdata = tempdata - 0.046; %account for diffraction by pure LB and antibiotics
    averages_downstreamQF{condition} = mean(tempdata'); %average OD for each timepoint
    error_downstreamQF{condition} = std(tempdata'); %standard deviation for each timepoint
end

t = 0:0.5:0.5*(length(plus15QF)-1);
figure
hold on
for r = 1:length(averages_downstreamQF)
    plot(t, averages_downstreamQF{r}, '-o')
    eb1 = errorbar(t, averages_downstreamQF{r}, error_downstreamQF{r});
    eb1.Color = [0 0 0];
    eb1.LineStyle = 'none';
    set(get(get(eb1, 'Annotation'), 'LegendInformation'), 'IconDisplayStyle', 'off');
end
xlabel('Time (Hrs)')
ylabel('OD_{600}')
h = legend('T7-15-QUAS-GFP + QF', 'T7-10-QUAS-GFP + QF', 'T7-5-QUAS-GFP + QF', 'No Plasmid');
set(h, 'Location', 'NorthWest');
title('Downstream Constructs with QF')

%% Supplemental Figure 6e
clear

ColB = [0.074	0.073	0.073
0.076	0.077	0.076
0.076	0.081	0.08
0.091	0.100	0.094
0.118	0.122	0.125
0.175	0.145	0.162
0.222	0.272	0.264
0.327	0.39	0.349
0.432	0.512	0.434
0.496	0.567	0.515
0.539	0.6	0.571
0.674	0.664	0.637
0.653	0.704	0.67
0.668	0.754	0.692
0.734	0.787	0.788
0.738	0.752	0.763
```

```
0.791   0.813   0.785
0.796   0.8 0.805
0.802   0.843   0.82
0.706   0.74    0.81
0.783   0.828   0.799];

T7LacO = [0.071 0.075   0.072
0.075   0.08    0.079
0.095   0.099   0.089
0.119   0.121   0.11
0.198   0.171   0.146
0.297   0.289   0.264
0.405   0.373   0.357
0.516   0.478   0.45
0.593   0.567   0.506
0.579   0.629   0.575
0.635   0.596   0.587
0.715   0.654   0.611
0.764   0.604   0.695
0.724   0.669   0.677
0.805   0.754   0.793
0.767   0.729   0.84
0.81    0.777   0.778
0.946   0.732   0.822
0.85    0.817   0.806
0.81    0.824   0.802
0.778   0.899   0.889];

BL21 = [0.099   0.104   0.096
0.103   0.104   0.096
0.137   0.136   0.121
0.189   0.213   0.179
0.267   0.277   0.256
0.397   0.465   0.362
0.457   0.47    0.468
0.574   0.564   0.537
0.617   0.642   0.67
0.66    0.641   0.62
0.698   0.712   0.732
0.736   0.756   0.764
0.801   0.804   0.841
0.883   0.871   0.836
0.876   0.887   0.882
0.918   0.924   0.915
0.982   0.978   0.894
0.994   1.004   0.993
0.973   1   0.996
0.955   1.062   1.031
1.119   1.14    1.049];

plus15QF = [0.072   0.073   0.07
0.072   0.074   0.073
0.076   0.073   0.075
0.077   0.077   0.079
```

```
0.083   0.083   0.083
0.098   0.101   0.099
0.109   0.12    0.135
0.174   0.167   0.154
0.213   0.276   0.283
0.34    0.352   0.351
0.396   0.441   0.551
0.553   0.536   0.509
0.566   0.562   0.523
0.541   0.555   0.622
0.65    0.596   0.588
0.647   0.632   0.724
0.601   0.606   0.63
0.632   0.635   0.625
0.738   0.61    0.702
0.705   0.721   0.735
0.721   0.71    0.731];

plus10 = [0.08  0.081   0.079
0.084   0.086   0.084
0.093   0.097   0.095
0.115   0.115   0.116
0.182   0.167   0.168
0.28    0.298   0.319
0.4 0.413   0.402
0.516   0.529   0.526
0.57    0.582   0.585
0.621   0.676   0.583
0.627   0.599   0.625
0.636   0.65    0.654
0.688   0.675   0.702
0.757   0.734   0.756
0.746   0.763   0.731
0.822   0.793   0.77
0.87    0.919   0.931
0.826   0.925   0.94
0.93    0.88    1.043
0.947   1.024   0.975
0.909   0.962   1.02];

random = {plus15QF, plus10, T7LacO, BL21, ColB};
% random = {ColB, T7LacO, BL21};

for r = 1:length(random)
    averages_random{r} = mean(random{r}'-0.046);
    error_random{r} = std(random{r}');
end

t = 0:0.5:(length(ColB)-1)*0.5;

figure
hold on
for r = 1:length(random)
    if r == 4
```

```
        plot(t, averages_random{r}, '-o', 'Color', [0.635 0.078 0.184])
        eb = errorbar(t, averages_random{r}, error_random{r});
        eb.Color = [0, 0, 0];
        eb.LineStyle = 'none';
        set(get(get(eb, 'Annotation'), 'LegendInformation'), 'IconDisplayStyle', 'off');
    else
        plot(t, averages_random{r}, '-o')
        eb = errorbar(t, averages_random{r}, error_random{r});
        eb.Color = [0, 0, 0];
        eb.LineStyle = 'none';
        set(get(get(eb, 'Annotation'), 'LegendInformation'), 'IconDisplayStyle', 'off');
    end
end

xlabel('Time (Hrs)')
ylabel('OD_{600}')
title('Growth Curves')
h = legend('T7-15-QUAS + QF', 'T7-10-QUAS', 'T7', 'No Plasmid', 'High pass filter');
set(h, 'Location', 'Best');
```

Supplementary Software Figure 8: Matlab code for generating all of the plots in Supplementary Figure 6.

Qplotcyt Function:

```
function graph = Qplotcyt(t, data, graphtitle, condition1, condition2)
% This function accepts data from a timed experiment experiment arranged in
% a matrix. With time across the top (i.e. column 1 is hr 1, col 2 is hr 2, etc.) The
% conditions are listed along each row in triplicate (condition1 is row
% 1-3,condition 2 is row 4-6)
% This function will create a plot with error bars depicting the standard
% deviation of the mean.
% Normalization is for the cytoflex flow cytometer in SMBB. Each
% data point is divided by the average value of T7-lacO-GFP (control) at
% hour 1.
% t is the time vector, i.e. the time points at which the data were
% collected (1, 2, 3, 4, ...)
% graphtitle, condition1, and condition2 must be entered as strings
% The output variable, graph, can be used to save the figure as a variable.

%Data from CytoFlex. Collected 1/27/20
T7LacO = [11295 24175   14078   4671    2056    1248    895 774 679 669
19170   19952   13918   5519    2750    1668    1347    1150    963 983
7428    20767   9792    4340    2256    1547    1338    1085    1085    913];

norm = mean(T7LacO(:,1)); % Divide results by this value to normalize data

%data normalization
data = data./norm;
T7LacO = T7LacO./norm;
stop = t(end);

I = mean(data(1:3, :));
II = mean(data(4:6, :));
III = mean(T7LacO(:, 1:stop));

% Determine standard deviation
n =1;
col = 1;
erI = zeros(1, stop);
while col <= stop
    erI(n) = std(data(1:3, col)); %error for I
    n = n+1;
    col = col +1;
end

n =1;
erII = zeros(1, stop);
for col = 1:stop
    erII(n) = std(data(4:6, col)); %error for II
    n = n+1;
end

n =1;
erIII = zeros(1, stop);
for col = 1:stop
    erIII(n) = std(T7LacO(:, col)); %error for II
    n = n+1;
```

```
end

bg = [III(:), I(:), II(:)]; %bar graph data

%create time vectors for data points
time_i = [];
time_ii = [];
time_iii = [];

for r = 1:length(t)
    temp = ones(1,3).*r - 0.225; %temporary vector
    time_iii = [time_iii, temp];
end

for r = 1:length(t)
    temp = ones(1,3).*r; %temporary vector
    time_i = [time_i, temp];
end

for r = 1:length(t)
    temp = ones(1,3).*r + 0.225; %temporary vector
    time_ii = [time_ii, temp];
end

time_points = [time_iii, time_i, time_ii]; %time vector for data_points

data_iii = T7LacO(:, :);
data_iii = data_iii(:)';
data_i = data(1:3, :);
data_i = data_i(:)';
data_ii = data(4:6, :);
data_ii = data_ii(:)';

data_points = [data_iii, data_i, data_ii];

graph = figure;
hold on
b = bar(t, bg, 'grouped');
b(1).FaceColor = [0.494, 0.184 0.556];
b(2).FaceColor = [0, 0.447, 0.741];
b(3).FaceColor = [0, 0.75, 0];
plot(time_points, data_points, '.k')
title(graphtitle, 'FontSize', 13)
xlabel('Time (hours)', 'FontSize', 12)
ylabel('Normalized GFP Fluorescence', 'FontSize', 12)
% ylim([0 8]);

%Add error bars
hold on
ebIII = errorbar(t-0.225, III, erIII); %error bar one. Centered on first group
ebIII.Color = [0, 0, 0];
ebIII.LineStyle = 'none';

ebI = errorbar(t, I, erI); %error bar two centered on second group
```

```
ebI.Color = [0 0 0];
ebI.LineStyle = 'none';

ebII = errorbar(t+0.225, II, erII); %error bar two centered on second group
ebII.Color = [0 0 0];
ebII.LineStyle = 'none';

h = legend('T7', condition1, condition2);
set(h, 'FontSize', 10);
hold off
end
```

Supplementary Software Figure 9: Matlab function for plotting the data in Figure 3c.

QplotSxP_new

```
function graph = QplotDxP_new(t, data, graphtitle, condition1, condition2)
% This function accepts data from a timed experiment experiment arranged in
% a matrix. With time across the top (i.e. column 1 is hr 1, col 2 is hr 2, etc.) The
% conditions are listed along each row in triplicate (condition1 is row
% 1-3,condition 2 is row 4-6)
% This function will create a plot with error bars depicting the standard
% deviation of the mean.
% Normalization is for the DxP flow cytometer in the Wintrobe building after it was
% calibrated.
% Each data point is divided by the average value of T7-lacO-GFP (control) at
% hour 1.
% t is the time vector, i.e. the time points at which the data were
% collected (1, 2, 3, 4, ...)
% graphtitle, condition1, and condition2 must be entered as strings
% The output variable, graph, can be used to save the figure as a variable.

% T7lacO data from callibrated DxP. Data from 11/8/19 and 11/5/19
T7LacO = [8739  12853   7568    4576    1471    963 748 1192    651 666
8488    12870   7102    2968    1615    1101    866 856 825 848
8901    12194   6330    2478    1373    835 625 625 583 529

1490    10843   6400    2840    1385    1007    771 656 663 588
2343    7954    3941    1402    746 599 536 456 433 404
2125    9674    4945    2207    1259    1034    954 967 956 882];

norm = mean(T7LacO(:,1)); % Divide results by this value to normalize data

%data normalization
data = data./norm;
T7LacO = T7LacO./norm;

I = mean(data(1:3, :));
II = mean(data(4:6, :));
III = mean(T7LacO(:, :));

% Determine standard deviation
n =1;
col = 1;
stop = t(end);
erI = zeros(1, stop);
while col <= stop
    erI(n) = std(data(1:3, col)); %error for I
    n = n+1;
    col = col +1;
end

n =1;
erII = zeros(1, stop);
for col = 1:stop
    erII(n) = std(data(4:6, col)); %error for II
    n = n+1;
end
```

```
n =1;
erIII = zeros(1, stop);
for col = 1:stop
    erIII(n) = std(T7LacO(:, col)); %error for II
    n = n+1;
end

bg = [III(:), I(:), II(:)]; %bar graph data
%create time vectors for data points
time_i = [];
time_ii = [];
time_iii = [];

for r = 1:length(t)
    temp = ones(1,6).*r - 0.225; %temporary vector
    time_iii = [time_iii, temp];
end

for r = 1:length(t)
    temp = ones(1,3).*r; %temporary vector
    time_i = [time_i, temp];
end

for r = 1:length(t)
    temp = ones(1,3).*r + 0.225; %temporary vector
    time_ii = [time_ii, temp];
end

time_points = [time_iii, time_i, time_ii]; %time vector for data_points

data_iii = T7LacO(:, :);
data_iii = data_iii(:)';
data_i = data(1:3, :);
data_i = data_i(:)';
data_ii = data(4:6, :);
data_ii = data_ii(:)';

data_points = [data_iii, data_i, data_ii];

graph = figure;
hold on
b = bar(t, bg, 'grouped');
b(1).FaceColor = [0.494, 0.184 0.556];
b(2).FaceColor = [0, 0.447, 0.741];
b(3).FaceColor = [0, 0.75, 0];
plot(time_points, data_points, '.k')
title(graphtitle, 'FontSize', 13)
xlabel('Time (hours)', 'FontSize', 12)
ylabel('Normalized GFP Fluorescence', 'FontSize', 12)
% ylim([0 8]);

%Add error bars
hold on
```

```
ebIII = errorbar(t-0.225, III, erIII); %error bar one. Centered on first group
ebIII.Color = [0, 0, 0];
ebIII.LineStyle = 'none';

ebI = errorbar(t, I, erI); %error bar two centered on second group
ebI.Color = [0 0 0];
ebI.LineStyle = 'none';

ebII = errorbar(t+0.225, II, erII); %error bar two centered on second group
ebII.Color = [0 0 0];
ebII.LineStyle = 'none';

h = legend('T7', condition1, condition2);
set(h, 'FontSize', 10);
hold off
end
```

Supplementary Software Figure 10: Matlab function to plot the data in Figure 3a and 3b.

QplotDxP:

```
function graph = QplotDxP(t, data, graphtitle, condition1, condition2)
% This function accepts data from a timed experiment experiment arranged in
% a matrix. With time across the top (i.e. column 1 is hr 1, col 2 is hr 2, etc.) The
% conditions are listed along each row in triplicate (condition1 is row
% 1-3,condition 2 is row 4-6)
% This function will create a plot with error bars depicting the standard
% deviation of the mean.
% Normalization is for the DxP flow cytometer in the Wintrobe building *before* it
% was newly calibrated.
% Each data point is divided by the average value of T7-lacO-GFP (control) at
% hour 1.
% t is the time vector, i.e. the time points at which the data were
% collected (1, 2, 3, 4, ...)
% graphtitle, condition1, and condition2 must be entered as strings
% The output variable, graph, can be used to save the figure as a variable.

% T7LacO data from DxP. Data from 7/31/19, 9/1/19, and 9/2/19
T7LacO = [3439  3687    1960    1007    625 459 381 376 383 390
3584    4096    2081    887 542 416 382 374 376 382
3649    3547    1570    703 430 381 381 375 385 402

4167    4215    1775    1014    742 493 478 461 498 522
4483    4738    2064    742 491 439 458 470 480 526
4014    3668    1301    550 415 382 404 443 490 530

3581    2898    1220    632 537 605 491 454 472 504
3453    2656    860 443 318 382 398 443 469 514
3221    2198    719 414 319 372 399 422 462 507];

norm = mean(T7LacO(:,1)); % Divide results by this value to normalize data

hours = t(end); %total number of hours for the experiment

%data normalization
data = data./norm;
T7LacO = T7LacO./norm;

I = mean(data(1:3, :));
II = mean(data(4:6, :));
III = mean(T7LacO(:, 1:hours));

% Determine standard deviation
n =1;
col = 1;
stop = t(end);
erI = zeros(1, stop);
while col <= stop
   erI(n) = std(data(1:3, col)); %error for I
   n = n+1;
   col = col +1;
end

n =1;
```

```
erII = zeros(1, stop);
for col = 1:stop
    erII(n) = std(data(4:6, col)); %error for II
    n = n+1;
end

n =1;
erIII = zeros(1, stop);
for col = 1:stop
    erIII(n) = std(T7LacO(:, col)); %error for II
    n = n+1;
end

bg = [III(:), I(:), II(:)]; %bar graph data
%create time vectors for data points
time_i = [];
time_ii = [];
time_iii = [];

for r = 1:length(t)
    temp = ones(1,9).*r - 0.225; %temporary vector
    time_iii = [time_iii, temp];
end

for r = 1:length(t)
    temp = ones(1,3).*r; %temporary vector
    time_i = [time_i, temp];
end

for r = 1:length(t)
    temp = ones(1,3).*r + 0.225; %temporary vector
    time_ii = [time_ii, temp];
end

time_points = [time_iii, time_i, time_ii]; %time vector for data_points

data_iii = T7LacO(:, 1:length(t));
data_iii = data_iii(:)';
data_i = data(1:3, :);
data_i = data_i(:)';
data_ii = data(4:6, :);
data_ii = data_ii(:)';

data_points = [data_iii, data_i, data_ii];

graph = figure;
hold on
b = bar(t, bg, 'grouped');
b(1).FaceColor = [0.494, 0.184 0.556];
b(2).FaceColor = [0, 0.447, 0.741];
b(3).FaceColor = [0, 0.75, 0];
plot(time_points, data_points, '.k')
title(graphtitle, 'FontSize', 13)
xlabel('Time (hours)', 'FontSize', 12)
```

```
ylabel('Normalized GFP Fluorescence', 'FontSize', 12)
% ylim([0 8]);

%Add error bars
hold on
ebIII = errorbar(t-0.225, III, erIII); %error bar one. Centered on first group
ebIII.Color = [0, 0, 0];
ebIII.LineStyle = 'none';

ebI = errorbar(t, I, erI); %error bar two centered on second group
ebI.Color = [0 0 0];
ebI.LineStyle = 'none';

ebII = errorbar(t+0.225, II, erII); %error bar two centered on second group
ebII.Color = [0 0 0];
ebII.LineStyle = 'none';

h = legend('T7', condition1, condition2);
set(h, 'FontSize', 10);
hold off
end
```

Supplementary Software Figure 11: Matlab function to plot the data in Figure 1a, 1b, 2a, 2b, and 2c.
