# Supplementary material for: Enhanced regulation of prokaryotic gene expression by a eukaryotic transcriptional activator
Source: Nat Commun. 2021 Jul 5;12:4109. doi: 10.1038/s41467-021-24434-9 (PMC8257575; doi:10.1038/s41467-021-24434-9)
Supplement: Supplementary file 4 — Reporting Summary [file 41467_2021_24434_MOESM4_ESM.pdf]

nature research

Corresponding author(s): Tara L. Deans

Last updated by author(s): 06/09/2021

Reporting Summary

Nature Research wishes to improve the reproducibility of the work that we publish. This form provides structure for consistency and transparency in reporting. For further information on Nature Research policies, see our Editorial Policies and the Editorial Policy Checklist.

Statistics

For all statistical analyses, confirm that the following items are present in the figure legend, table legend, main text, or Methods section.

n/a	Confirmed	
☐	☒	The exact sample size (n) for each experimental group/condition, given as a discrete number and unit of measurement
☐	☒	A statement on whether measurements were taken from distinct samples or whether the same sample was measured repeatedly
☐	☒	The statistical test(s) used AND whether they are one- or two-sided<br>*Only common tests should be described solely by name; describe more complex techniques in the Methods section.*
☒	☐	A description of all covariates tested
☐	☒	A description of any assumptions or corrections, such as tests of normality and adjustment for multiple comparisons
☐	☒	A full description of the statistical parameters including central tendency (e.g. means) or other basic estimates (e.g. regression coefficient) AND variation (e.g. standard deviation) or associated estimates of uncertainty (e.g. confidence intervals)
☒	☐	For null hypothesis testing, the test statistic (e.g. F, t, r) with confidence intervals, effect sizes, degrees of freedom and P value noted<br>*Give P values as exact values whenever suitable.*
☒	☐	For Bayesian analysis, information on the choice of priors and Markov chain Monte Carlo settings
☒	☐	For hierarchical and complex designs, identification of the appropriate level for tests and full reporting of outcomes
☒	☐	Estimates of effect sizes (e.g. Cohen's d, Pearson's r), indicating how they were calculated

Our web collection on statistics for biologists contains articles on many of the points above.

Software and code

Policy information about availability of computer code

Data collection	Flow cytometry data were collected using CytExpert (version 2.4.0.28, Beckman Coulter). Optical density was measured using a Synergy HTX Reader (Biotek).
Data analysis	The geometric mean of each sample was calculated via FlowJo (version 10.6.1, FlowJo LLC) or Flowing Software (Cell Imaging Core, Turkey Centre for Biotech). The average of geometric means and standard deviation was calculated using Excel (2016, Microsoft). All plots were created via MATLAB (R2019b, The MathWorks, Inc.).

For manuscripts utilizing custom algorithms or software that are central to the research but not yet described in published literature, software must be made available to editors and reviewers. We strongly encourage code deposition in a community repository (e.g. GitHub). See the Nature Research guidelines for submitting code & software for further information.

Data

Policy information about availability of data

All manuscripts must include a data availability statement. This statement should provide the following information, where applicable:

- Accession codes, unique identifiers, or web links for publicly available datasets
- A list of figures that have associated raw data
- A description of any restrictions on data availability

All data needed to evaluate the conclusions in the paper are present in the paper and/or in the Supplementary Materials. Additional data are available from the corresponding author upon reasonable request. All MATLAB code is available in the Supplementary Software File. All plasmids in this study will be deposited in Addgene, which are listed in Supplementary Table 3.

Field-specific reporting

Please select the one below that is the best fit for your research. If you are not sure, read the appropriate sections before making your selection.

☒ Life sciences ☐ Behavioural & social sciences ☐ Ecological, evolutionary & environmental sciences

For a reference copy of the document with all sections, see nature.com/documents/nr-reporting-summary-flat.pdf

Life sciences study design

All studies must disclose on these points even when the disclosure is negative.

Sample size	Flow cytometry was used to analyze at least 10,000 cells per sample. Each condition was performed in triplicate with biological replicates. Sample size was determined by a priori analysis using G*Power software (version 3.1.9.6, University of Kiel) using data from preliminary gene expression experiments. The sample size of 3 for each experimental condition was calculated using a two-tailed t-test at 95% confidence. This aligns with previous work in the field, as a sample size of 3 is typical for gene expression experiments.
Data exclusions	Data were not excluded.
Replication	All experiments were conducted in triplicate with biological replicates. Several constructs were tested on additional days with results consistent with those reported here. All observations are consistent with the results reported herein.
Randomization	Samples were not randomized. Cultures were treated with inducers as indicated in the Methods section. Covariates were minimized by subjecting all samples from conditions which were compared to each other to the same experimental conditions: all samples were transformed at the same time, grown in the same media, and induced at the same optical density.
Blinding	Investigators were not blind to experimental conditions. This was not possible, as the experimenters determined the experimental conditions.

Reporting for specific materials, systems and methods

We require information from authors about some types of materials, experimental systems and methods used in many studies. Here, indicate whether each material, system or method listed is relevant to your study. If you are not sure if a list item applies to your research, read the appropriate section before selecting a response.

Materials & experimental systems

n/a	Involved in the study	
☒	☐	Antibodies
☒	☐	Eukaryotic cell lines
☒	☐	Palaeontology and archaeology
☒	☐	Animals and other organisms
☒	☐	Human research participants
☒	☐	Clinical data
☒	☐	Dual use research of concern

Methods

n/a	Involved in the study	
☒	☐	ChIP-seq
☐	☒	Flow cytometry
☒	☐	MRI-based neuroimaging

Flow Cytometry

Plots

Confirm that:

☒ The axis labels state the marker and fluorochrome used (e.g. CD4-FITC).

☒ The axis scales are clearly visible. Include numbers along axes only for bottom left plot of group (a 'group' is an analysis of identical markers).

☒ All plots are contour plots with outliers or pseudocolor plots.

☒ A numerical value for number of cells or percentage (with statistics) is provided.

Methodology

Sample preparation	Bacterial cultures were diluted 1:400 in phosphate-buffered saline solution. A CytoFLEX S or DXP flow cytometer was used to measure GFP expression. 10,000 bacteria were measured for each sample via gating in the forward scatter (FSC) vs. side scatter plot (SSC).
Instrument	CytoFLEX S flow cytometer (Beckman Coulter), DXP flow cytometer (Cytek)

Software	Flow cytometry data were collected using CytExpert (version 2.4.0.28, Beckman Coulter). The geometric mean of each sample was calculated via FlowJo (version 10.6.1, FlowJo LLC). The average of geometric means and standard deviation was calculated using Excel (2016, Microsoft) and plotted via MATLAB (R2019b, MathWorks).
Cell population abundance	The cells were not sorted.
Gating strategy	Cells were gated in the FSC vs. SSC plot. A polygonal gate was used to capture the bacterial population. This gating strategy is shown in Supplemental Figure 7.

☒ Tick this box to confirm that a figure exemplifying the gating strategy is provided in the Supplementary Information.
